# Bidirectional relationship between burnout and perceived work ability: Evidence from a two‐wave study among teachers

**DOI:** 10.1111/aphw.70075

**Published:** 2025-09-09

**Authors:** Petr Hlado, Tomáš Lintner, Libor Juhaňák, Klara Harvankova

**Affiliations:** ^1^ Department of Educational Sciences Masaryk University, Faculty of Arts Czech Republic; ^2^ Institute of Psychology of the Czech Academy of Sciences Czech Republic; ^3^ Institute of Social Sciences of the Slovak Academy of Sciences Slovakia

**Keywords:** burnout, conservation of resources theory, cross‐lagged panel modeling, job demands‐resources model, perceived work ability, work ability

## Abstract

Burnout and perceived work ability (PWA) are critical factors influencing teachers' professional well‐being and effectiveness. The potential bidirectional relationship between these constructs remains underexplored, particularly in primary and lower secondary school teachers. This study examines the reciprocal relationship between burnout and PWA among teachers over time, using the job demands‐resources (JD‐R) model and the conservation of resources (COR) theory. A two‐wave longitudinal study was conducted among 853 Czech primary and lower secondary school teachers. Data were collected via web‐based questionnaires at two time points. Bayesian cross‐lagged panel modeling (CLPM) was applied to analyze the bidirectional effects of burnout and PWA while controlling demographic variables. Burnout significantly predicted lower PWA, supporting the JD‐R model's health impairment process. Contrary to expectations, higher PWA was associated with increased burnout, suggesting that PWA may not function as a protective factor in the teaching context, but rather as a risk factor increasing vulnerability to strain. Post hoc analyses indicated that burnout's detrimental influence on PWA emerged through several coherent facet‐to‐facet pathways, while in the reverse direction, only higher PWA in the teaching organization predicted later physical exhaustion. The study clarifies the directionality of associations between burnout and PWA, contributing to theory development and offering implications for interventions.

## INTRODUCTION

Teachers play a pivotal role in students' development and the overall educational process. However, many educational systems are currently experiencing significant teacher shortages, primarily due to a growing gap between the large number of teachers retiring or leaving the profession and the insufficient influx of graduates from teacher education programs (European Commission, 2023; UNESCO, 2024; Van Den Borre et al., 2021). These shortages place considerable strain on the remaining teaching workforce, often requiring teachers to assume additional responsibilities and manage increased workloads (European Commission, 2023; UNESCO, 2024).

Teachers are often faced with excessive job demands on their energy, strength, and resources, which can exceed their capacity to manage them effectively (Agyapong et al., 2022; Hakanen et al., 2006; Iriarte Redín & Erro‐Garcés, 2020; Mijakoski et al., 2022). Previous research suggests that high job demands and prolonged overload hinder teachers' daily functioning, expose them to various risks that can impair their performance, and diminish the capacity to meet the demands of their work (Hlado et al., 2025a), thereby preventing them from continuing in the profession (Droogenbroeck & Spruyt, 2014). Therefore, it is crucial to develop and implement targeted support strategies aimed at enhancing teachers' capacity across all age groups to effectively meet job demands and improve retention within the profession. The potential strategies involve preventing teacher burnout and enhancing work ability (Hlado et al., 2025b). These constructs are widely recognized as key factors in evaluating teachers' capacity to meet job demands, as they reflect the energy available as well as the resources teachers can dedicate to work task completion (Mijakoski et al., 2022; Schaufeli & Taris, 2014).

Teacher burnout is a psychological syndrome resulting from prolonged exposure to chronic occupational stressors. In the present study, burnout is defined in accordance with the Shirom–Melamed framework as a state of physical fatigue, emotional exhaustion, and cognitive weariness (Shirom, 2003a; Shirom & Melamed, 2006). This conceptualization is grounded in Hobfoll's conservation of resources theory (COR; Hobfoll, 1989, 2001), which posits that these three components represent interrelated manifestations of a progressive depletion of energetic resources, thereby impairing an individual's capacity to function effectively in the workplace.

Burnout represents a significant psychological concern within the teaching profession (Iancu et al., 2018; Ozamiz‐Etxebarria et al., 2023; Seibt et al., 2009). Research shows that teachers experience higher levels of burnout than most other professions (Droogenbroeck & Spruyt, 2014). This phenomenon is likely attributable to a wide range of daily job demands and stressors they face (Hlado & Harvankova, 2024; Madigan & Kim, 2021; Ptáček et al., 2019). For teachers who remain in their roles, burnout results in reduced productivity and teaching effectiveness (Wolf et al., 2015). It also contributes to lower job commitment, higher absenteeism, and increased turnover intentions, making it a key factor in teacher attrition (Agyapong et al., 2022; Billingsley & Bettini, 2019; Madigan & Kim, 2021; Salvagioni et al., 2017).

In recent years, an increasing number of meta‐analyses and systematic reviews have focused on interventions targeting teacher burnout (Agyapong et al., 2023; Avola et al., 2025; Hidajat et al., 2023a) and on identifying its key determinants (Gooden et al., 2023; Liu et al., 2022; Menon et al., 2024; Mijakoski et al., 2022; Sohail et al., 2023). This growing body of research emphasizes the interplay of organizational and individual factors in burnout development. At the organizational level, burnout is linked to adverse working conditions, including excessive workload, limited autonomy, classroom disruptions, and unsupportive school environments. Interpersonal stressors, such as poor collegial relationships, lack of support, and workplace bullying, further increase risk. At the individual level, burnout is associated with personality traits (e.g., neuroticism, rejection sensitivity), low self‐efficacy, negative cognitive appraisals, maladaptive motivation, and limited emotional regulation and psychological resilience. Although extensive research has addressed the antecedents and consequences of teacher burnout, it has predominantly relied on cross‐sectional and unidirectional research designs.

Work ability (WA) is defined as an individual's capacity to perform required work tasks and manage job demands within their current occupational role (Gould, 2008; Ilmarinen et al., 1997; Tuomi, 2001). Recently, the construct has been differentiated into objective and perceived WA (Brady et al., 2020). Objective WA is based on the assessment of employees' health and functional limitations. Perceived work ability (PWA) represents the subjective perception of one's WA (McGonagle et al., 2015). The present study examines PWA among teachers, conceptualized as the subjective evaluation of physical and mental capacity to meet job requirements and manage the diverse physical, cognitive, interpersonal, emotional, and organizational demands inherent in contemporary teaching roles (Hlado & Harvankova, 2024; McCarthy et al., 2024; McGonagle et al., 2015).

Ilmarinen et al. (2005) highlights the importance of high WA in extending individuals' working lives and reducing the risk of early retirement. In contrast, low WA is associated with various adverse employment outcomes, including diminished work performance and productivity (Van Den Berg et al., 2009), absenteeism (Alavinia et al., 2009; Sell, 2009), and an increased likelihood of leaving the profession prematurely or retiring early (McCarthy et al., 2024; McGonagle et al., 2015; Vertanen‐Greis et al., 2022). From this perspective, WA in the educational context may represent a risk factor, as research indicates a progressive reduction of WA across the work‐life span (Cadiz et al., 2019; Van Den Berg et al., 2009).

Despite growing efforts to theoretically anchor PWA within the job demands‐resources model (JD‐R; Bakker et al., 2005, 2007; Bakker & Demerouti, 2007, 2017; Demerouti & Bakker, 2011), its position within this framework remains ambiguous. Specifically, it is unclear whether PWA should be conceptualized as a health‐related resource that mitigates the risk of burnout (Airila et al., 2014; Viotti et al., 2019), or as a work‐related outcome that deteriorates in response to prolonged job stressors (Hlado et al., 2025a; Hlado & Harvankova, 2024; Schaufeli, 2017).

Likewise, empirical evidence regarding the association between burnout and WA remains limited, and WA has been largely overlooked in recent meta‐analyses and systematic reviews (e.g., Gooden et al., 2023; Liu et al., 2022; Menon et al., 2024; Mijakoski et al., 2022; Sohail et al., 2023). According to the conceptual integration model of WA proposed by Cadiz et al. (2019), burnout may serve as either an antecedent or an outcome of WA. However, longitudinal evidence for this proposition remains limited. A notable contribution is the cross‐lagged panel study by Viotti et al. (2019), which reported that higher WA was linked to greater enthusiasm toward the job and lower exhaustion, but not cynicism. Moreover, none of the three burnout subdimensions significantly predicted WA. These findings diverge from the assumptions of the conceptual integration model (Cadiz et al., 2019) and underscore the need for theoretical reflection and systematic investigation of the bidirectional relationship and temporal dynamics between burnout and PWA among teachers.

While the study by Viotti et al. (2019) provides valuable insights relevant to the present research, it also has limitations that warrant further investigation. To address these limitations, the current study adopts an alternative theoretical framework and employs a distinct methodological design. Whereas Viotti et al. (2019) primarily relied on the COR theory (Hobfoll, 1989, 2001), the present study integrates two complementary perspectives: the job demands‐resources model (JD‐R; Bakker et al., 2005, 2007; Bakker & Demerouti, 2007, 2017; Demerouti & Bakker, 2011) and the COR theory (Hobfoll, 1989, 2001).

Furthermore, the findings reported by Viotti et al. (2019) were based on a specific professional cohort, namely preschool teachers responsible for the care of children up to three years of age. The job demands inherent to this professional role differ substantially from those encountered by primary and lower secondary school teachers (Mijakoski et al., 2022). Teachers in primary and lower secondary education encounter distinct cognitive, emotional, and instructional demands related to curriculum implementation, classroom management, student assessment, and responding to the developmental and psychosocial needs of students in middle childhood and early adolescence. These contextual distinctions raise important questions regarding the generalizability of previous findings to other educational settings. Accordingly, examining the relationships between burnout and PWA among primary and lower secondary school teachers is crucial for determining whether these associations are also applicable to this professional group.

The present study also addresses another limitation identified in the work of Viotti et al. (2019), namely the measurement of PWA using the short version of the Work Ability Index (WAI; Tuomi et al., 2017). Although the WAI is widely employed in occupational health research (Cadiz et al., 2019; Magnavita et al., 2024), it has been criticized for lacking a clearly defined nomological network and for limited theoretical integration with related constructs (Brady et al., 2020). Concerns have likewise been raised regarding its construct validity, given insufficient evidence for both convergent and discriminant validity (Brady et al., 2020; Cadiz et al., 2019; McCarthy et al., 2024). Furthermore, the WAI assesses PWA through a small set of items that share conceptual overlap with constructs such as self‐efficacy and employability, and prior studies have reported partial overlap between WAI and burnout measures (Hlado & Harvankova, 2024; Mäkelä et al., 2015). To address these limitations, the present study examines the bidirectional relationship between burnout and PWA among teachers using a theoretically grounded, profession‐specific instrument designed to assess teachers' perceived physical and mental capacity to meet the multifaceted demands of their work, including lesson organization, classroom management, collegial collaboration, handling challenging situations, and fulfilling non‐teaching responsibilities (Hlado et al., 2025a).

In contrast to Viotti et al. (2019), who conceptualized burnout through three dimensions—psychological exhaustion, enthusiasm toward the job, and cynicism—and measured it using the Spanish Burnout Inventory (SBI; Guidetti et al., 2018), the present study adopts a resource‐based perspective grounded in the COR theory and operationalizes burnout with the Shirom–Melamed Burnout Inventory (SMBI; Ptáček et al., 2017; Shirom & Melamed, 2006). While the SBI assesses one energetic dimension (exhaustion) together with two motivational–attitudinal aspects of burnout (enthusiasm and cynicism), the SMBI is designed to capture exclusively the depletion of physical, emotional, and cognitive energy resources, thereby excluding behavioral or attitudinal components such as enthusiasm, cynicism, or disengagement. This conceptual orientation makes the SMBI suitable for examining its relationship with PWA among teachers, as it more directly reflects the erosion of energy resources essential for sustaining WA. By emphasizing the multidimensional depletion of energy required to meet job demands, the SMBI aligns closely with models positing that PWA depends on the availability of sufficient reserves to cope with job demands. This conceptual fit enhances the instrument's sensitivity to early changes in energy levels that may precede or accompany declines in PWA, thereby providing a robust basis for investigating how burnout affects teachers' capacity to fulfill their occupational responsibilities.

As such, this study offers an original contribution that goes beyond merely replicating the work of Viotti et al. (2019). While differences in operationalization must be acknowledged, we believe that our approach nonetheless provides valuable insights that complement and extend the existing literature. First, we investigate the bidirectional relationship between burnout and PWA using a two‐wave longitudinal design and Bayesian cross‐lagged panel modeling. This approach provides dynamic insights into their reciprocal effects over time and helps clarify the conceptual role of PWA within the JD‐R model. Second, we extend empirical knowledge by focusing on primary and lower secondary school teachers—a large yet understudied population facing complex curricular responsibilities, diverse student needs, and institutional challenges that often exceed those encountered by early childhood educators studied in previous research (Hakanen et al., 2006; Hlado & Harvankova, 2024; Kyriacou, 2001; Mäkelä et al., 2015; Mijakoski et al., 2022). Third, following the findings of Brady et al. (2020), our assessment of PWA incorporates contextual workplace factors, specifically teachers' capacity to meet the unique job demands of their daily professional practice. By grounding the study in the JD‐R model (Bakker et al., 2005, 2007; Bakker & Demerouti, 2007, 2017; Demerouti & Bakker, 2011) and the COR theory (Hobfoll, 1989, 2001), we also contribute to the theoretical refinement of the PWA construct, particularly by examining its position within a nomological network of occupational health. This dual‐theory approach enables a comprehensive conceptualization of PWA, framing it both as a health‐based resource and as a work‐related outcome. Finally, by identifying how burnout and PWA mutually influence each other, the present research provides evidence‐based entry points for targeted interventions.

## THE EFFECT OF TEACHER BURNOUT ON PERCEIVED WORK ABILITY

According to the JD‐R model (Bakker et al., 2005, 2007; Bakker & Demerouti, 2007, 2017; Demerouti & Bakker, 2011), job characteristics trigger two psychological processes: a motivational process supported by job resources and a health impairment process driven by job demands. The latter is especially relevant for teachers, whose work is characterized by persistent physical, cognitive, emotional, and organizational demands (Hlado & Harvankova, 2024). In the JD‐R model, prolonged high job demands gradually drain mental and physical reserves, leading to energy exhaustion and teacher burnout (Hakanen et al., 2006; Schaufeli & Taris, 2014). Burnout, in turn, contributes not only to the deterioration of teachers' health (Hlado et al., 2025b) but also to a range of adverse work‐related outcomes (Madigan et al., 2023; Salvagioni et al., 2017). Although the JD‐R model does not explicitly conceptualize PWA as a core construct, diminished WA is theorized within the model as a consequence of the health impairment process (Schaufeli, 2017). This mechanism was explored and described in more detail by Hlado and Harvankova (2024) in their qualitative study among upper secondary school teachers. When teachers perceived their job demands as excessive or unmanageable or faced barriers to meeting them, this typically triggered job‐related stress. If left unaddressed, such stress leads to fatigue, burnout, and physical as well as mental health problems, making it difficult or even impossible for teachers to fulfill their professional responsibilities. Building on this, we conceptualize PWA in the present study as a work‐related outcome within the health impairment process of the JD‐R model (Schaufeli, 2017).

Research employing a quantitative approach across various professions has shown that burnout is a key predictor of diminished WA. For instance, studies among physicians (Debets et al., 2022), nurses (Hatch et al., 2018), and food manufacturing workers (Arandjelovic et al., 2010) have shown a negative impact of burnout on WA. Comparable findings were reported in research on copper and nickel miners (Sun et al., 2020) and workers experiencing high levels of job‐related stress (Pranjic & Bilic, 2014). Furthermore, emotional exhaustion, cynicism, and reduced personal accomplishment, the core dimensions of burnout, were identified as predictors of low WA among biosafety laboratory staff during the COVID‐19 pandemic (Lu et al., 2021). In the teaching profession, burnout has been found to have a negative impact on WA across different age groups. Research among female grammar school teachers (Seibt et al., 2009), primary and lower secondary school teachers (Hlado et al., 2025a), and upper secondary school teachers (Hlaďo et al., 2020) has shown that burnout is a predictor of lower WA. Overall, research suggests that burnout poses a substantial risk to WA across various professions, including teaching. However, previous studies were predominantly based on cross‐sectional designs and assessed objective WA using the WAI, which limits the generalizability of their findings to the effect of burnout on teachers' PWA. Based on the JD‐R model and empirical evidence, we propose:

Hypothesis 1 Higher burnout predicts lower PWA among teachers over time.

## THE EFFECT OF PERCEIVED WORK ABILITY ON TEACHER BURNOUT

The revised JD‐R model (Bakker & Demerouti, 2007; Taris et al., 2017) highlights the critical role of personal resources such as hope, intrinsic motivation, optimism, resilience, self‐efficacy, and value orientation in promoting employee well‐being and adaptive functioning at work. Personal resources are considered essential for buffering the detrimental effects of job demands on strain and burnout (Schaufeli & Taris, 2014), thereby mitigating the health impairment process by reducing the risk of burnout (Taris et al., 2017).

However, PWA does not align with the conceptualization of personal resources as articulated in the JD‐R model. According to Schaufeli and Taris (2014), personal resources are defined as psychological characteristics or aspects of the self that are generally associated with resilience and reflect an individual's ability to control and effectively influence their environment. In contrast, PWA refers to an individual's perceived capacity to meet externally imposed job demands, rather than to internal dispositions such as self‐regulation, agency, or resilience. As suggested by Airila et al. (2014) and Viotti et al. (2019), PWA may be conceptualized as a health‐related resource. However, the position of such a resource within the JD‐R framework remains ambiguous and underdeveloped. Accordingly, we argue that the JD‐R model offers limited explanatory power for understanding the role of PWA in the development of burnout. From this perspective, the COR theory provides a more conceptually coherent framework for capturing this dynamic.

The COR (Hobfoll, 1989, 2001) theory explains how individuals regulate energy by striving to preserve, protect, and accumulate resources. Individuals strategically invest their resources to achieve goals, sustain gains, and protect themselves from loss. Resources typically cluster and accumulate over time, forming resource caravans—reinforcing cycles in which a strong resource base facilitates further resource acquisition (Hobfoll, 2002). Within work contexts, resources enable employees to meet job demands or reduce psychological strain, while their aggregation contributes to positive work outcomes (Hobfoll & Shirom, 2001). Conversely, risk factor caravans represent constellations of hazards that not only deplete existing resources but also undermine individuals' ability to replenish them, thereby exacerbating vulnerability (Holmgreen et al., 2017). Moreover, resource loss tends to trigger cycles of further resource depletion, which accelerate in both magnitude and speed. Resource loss tends to escalate through loss cycles, in which each depletion weakens an individual's capacity to cope with further stress, increasing the risk of exhaustion. When resources are lost, depleted, or cannot be secured despite effort, negative consequences such as psychological stress, fatigue, and burnout often follow (Hakanen et al., 2006; Hobfoll, 2001, 2002; Shirom, 2003b).

As previously noted, PWA can be conceptualized as health‐related resources (Airila et al., 2014; Viotti et al., 2019). Therefore, teachers with higher PWA are expected to be more effective in maintaining resource gain and less susceptible to loss. Conversely, teachers with lower PWA are at greater risk of resource depletion. As noted by Viotti et al. (2017), reduced PWA can act as a trigger for a loss spiral, leading to an imbalance between the job demands placed on teachers and the personal resources that individuals can rely on to meet those demands. The effort to compensate for limited PWA disrupts the teacher's energy balance and is likely to be accompanied by physical or mental fatigue, which may result in teacher burnout (Hlado & Harvankova, 2024; McGonagle et al., 2022a; Viotti et al., 2017).

From an empirical perspective, there is evidence indicating that WA may negatively predict burnout. However, it is crucial to acknowledge that the body of research exploring this relationship remains limited. A longitudinal study by Viotti et al. (2017) found that PWA predicts burnout among early childhood educators over a one‐year period. PWA was found to be more strongly linked to job enthusiasm than to emotional exhaustion. Moreover, no significant relationship was observed between PWA and cynicism. In a cross‐sectional study, Converso et al. (2021) identified lower levels of WA as a predictor of heightened emotional exhaustion and depersonalization among nurses. The potential impact of WA on burnout is further supported by meta‐analytic findings from Brady et al. (2020), who suggest that WA may serve as a mediating pathway through which workplace conditions influence psychological strain. To our knowledge, no other studies have investigated this direction of the relationship between WA and burnout. The absence of such research is further supported by several systematic reviews on the predictors of occupational burnout, none of which have addressed this specific relationship (e.g., Gooden et al., 2023; Liu et al., 2022; Menon et al., 2024; Mijakoski et al., 2022; Sohail et al., 2023). Thus, there is a need for longitudinal research that investigates the impact of PWA on burnout, particularly among primary and lower secondary school teachers. In this light, we hypothesize:

Hypothesis 2 Lower PWA predicts higher burnout levels among teachers over time.

## METHODS

### Data collection and participants

We conducted a two‐wave study among Czech primary (ISCED 1) and lower secondary (ISCED 2) school teachers. We compiled a database of schools in two regions of the Czech Republic—the South Moravian Region and the Vysočina Region, with nearly 15,000 teachers employed in these schools at the time of the data collection planning. We contacted a total of 352 schools via email with an invitation to participate in a longitudinal mixed‐methods study. We obtained consent to participate from 44 schools, and the study draws on data from the two waves of teacher questionnaires. We collected the data using a web‐based questionnaire.

The first wave (T1) of data collection took place during the 2023/24 school year, in October and November 2023. We chose the timing of data collection intentionally. In the Czech Republic, the school year begins in early September, when teachers return to their duties after two months of summer holidays. Therefore, we scheduled the data collection for the fall period, as teachers were already engaged in their regular teaching responsibilities but were not yet experiencing the work‐related fatigue and weariness commonly experienced in the months leading up to the summer holidays. Participation in the research was voluntary. At T1, the sample consisted of 853 teachers (86.1% female). Participants' ages ranged from 22 to 76 years, with a mean age of 45.9 years (*SD* = 10.8). On average, the teachers had 19.3 years of experience in the teaching profession (*SD* = 12.1). The sample included teachers from a range of subjects, such as languages, mathematics, natural sciences (biology, chemistry, physics), physical education (PE), and the arts.

The second wave of data collection (T2) took place during the 2024/25 school year, from November to December 2024, approximately 12 months from the first measurement point. At T2, 481 teachers (87.9% females) completed the survey. Participants' ages ranged from 23 to 68 years, with a mean age of 46.7 years (SD = 9.9), and they had an average of 20.2 years (SD = 11.6) of teaching experience. The attrition rate was 43.6%.

### Missing data treatment

We assumed that the data were missing at random (MAR) and addressed the issue of missingness through multiple imputation using the mice package in R (R Core Team, 2023) with predictive mean matching. This method is advantageous as it ensures that imputed values are realistic by selecting observed values from the dataset that are most similar to the predicted value. We imputed 20 separate datasets. To get an overall model result from the imputed datasets, we pooled full posterior distributions from Bayesian modeling described later.

Given the relatively high level of missing data due to a 50% response rate of teachers at T2, we carefully considered how to handle missingness. For our primary analysis, we opted against listwise deletion of teachers who did not participate at T2, as this would have resulted in a significant loss of valuable information regarding the relationships between variables at T1, where a larger sample was available. Instead, we performed the multiple imputation procedure to retain statistical power and preserve the integrity of the dataset. To assess the robustness of our findings, we conducted a sensitivity analysis using the non‐imputed data, including only the teachers who participated at both time points. The results from the non‐imputed data yielded the same substantial conclusions as the imputed ones; hence, we consider the imputation not to have significantly changed the results. The results of this sensitivity analysis are provided in Supplementary Tables S1 and S2.

### Measures

We assessed **PWA among teachers** using the Teacher Work Ability Scale (TWAS; Hlado et al., 2025). The TWAS measures teachers' perceived physical and mental capacity to meet various job demands within the teaching profession. It comprises 21 items rated on a 7‐point Likert scale (1 = low, 7 = high) and has a 5‐factor structure consisting of the following dimensions: teaching organization (4 items, an example item: “Organize team group work of students”), instructional management (5 items, an example item: “Keep students' attention in lesson”), teacher‐staff interaction (4 items, an example item: “Receive feedback from superiors and colleagues”), navigating difficult situations (3 items, an example item: “Address problematic situations with students”), and non‐teaching responsibilities (5 items, an example item: “Supervise students outside the classroom”). The validation study confirmed the five‐factor structure of the TWAS, demonstrated excellent internal consistency across dimensions (Cronbach's α ranging from .85 to .94), and showed strong convergent and discriminant validity. In the present study, the TWAS demonstrated high internal consistency, with Cronbach's α of .95 at T1 and .96 at T2, and ranging from .84 to .94 across the dimensions. We conducted a second‐order confirmatory factor analysis (CFA) to confirm the five‐factor structure of the TWAS. The results of the CFA at both T1 (χ^2^ |184| = 1204.1, *p* < .01, CFI = .92, TLI = .91, RMSEA = .08, SRMR = .05) and T2 (χ^2^|184| = 702.2, *p* < .01, CFI = .94, TLI = .93, RMSEA = .08, SRMR = .05) showed a good fit. Detailed scales' internal consistency and fit indices measures are available as Supplementary Table S3.

We measured **burnout** by the Czech version of the *Shirom‐Melamed Burnout Questionnaire* (SMBQ, Ptáček et al., 2017). SMBQ is a 14‐item inventory consisting of three subscales that measure physical exhaustion, cognitive weariness, and emotional exhaustion. The SMBQ items were measured on a 7‐point Likert‐type scale with response options ranging from 1 (*never or almost never*) to 7 (*always or almost always*). A high score indicates high burnout. Evidence from the Czech validation study (Ptáček et al., 2017) shows excellent internal consistency and supports the expected three‐factor structure with high factor loadings and good model fit. These findings confirm that the Czech SMBQ is a reliable and valid tool for assessing burnout in working populations. In the present study, Cronbach's α for the overall SMBQ was .93 at both T1 and T2 and ranged from .88 to .93 for the subscales. At both T1 (χ^2^ |73| = 413.6, *p* < .01, CFI = .96, TLI = .95, RMSEA = .07, SRMR = .04) and T2 (χ^2^|74| = 338.4, *p* < .01, CFI = .95, TLI = .94, RMSEA = .09, SRMR = .05), the CFA showed a good fit. Detailed scales' internal consistency and fit indices measures are available as Supplementary Table S3.

While we collected the study's primary variables (i.e., burnout and PWA) at both time points, we measured the control variables (e.g., gender, age, and years of practice) only at T1.

### Bayesian cross‐lagged panel model

To address our research question and test our hypotheses, we applied a Bayesian CLPM using the *blavaan* package (Merkle et al., 2021; Merkle & Rosseel, 2018). We chose the CLPM approach because it enables the examination of the bi‐directional nature of relationships between burnout and PWA, while controlling for an array of teachers' characteristics. The CLPM comprises three key types of relationships: (1) cross‐sectional, which capture contemporaneous associations between variables at a given time point; (2) stability, which assesses the autoregressive effects of variables over time; and (3) cross‐lagged relationships, which allow us to evaluate directional influences between variables across time points.

We adopted a Bayesian approach to the CLPM primarily for its advantages in handling complex model structures and moderate sample sizes, which characterize the present study. Specifically, Bayesian estimation offers greater numerical stability in model convergence compared to frequentist maximum likelihood estimation, particularly in structurally saturated cross‐lagged panel models with multiple predictors, outcomes, and control variables. In addition, Bayesian methods allow for more intuitive interpretation of parameter uncertainty: rather than relying on p‐values, the results are expressed as posterior distributions, which enable direct probabilistic statements about the direction and magnitude of effects.

Based on the posterior distributions, we calculated the posterior means (PMs), representing the central tendency of the effects, the 95% credible intervals (CrIs), indicating the range within which the true effect is likely to fall with a specified level of certainty, and the Probability of Direction (PD) index. The PD index quantifies the certainty of an effect's direction (positive or negative) on a scale from 0 to 1, providing a measure of the effect's existence. Mathematically, it is defined as the proportion of the posterior distribution that satisfies the specified hypothesis (Makowski, Ben‐Shachar, Chen, & Lüdecke, 2019). We calculated the metrics in *bayestestR* (Makowski, Ben‐Shachar, & Lüdecke, 2019).

For this analysis, we specified non‐informative priors to guide the estimation process without imposing any assumptions. We estimated the model using five Markov Chain Monte Carlo (MCMC) chains, each with a burn‐in period of 1,000 iterations, followed by 5,000 post‐burn‐in samples per chain. We assessed the model convergence using the rank‐based (Vehtari et al., 2021) Gelman‐Rubin diagnostic (*R̂*; Gelman & Rubin, 1992), and convergence was deemed acceptable if *R̂* was less than 1.01 for all parameters. This threshold indicates that the chains mixed well and reached a stable distribution, ensuring reliable posterior estimates. We assessed the model fit indices using established thresholds for acceptable fit, the Bayesian Gamma Hat ($\hat{\Gamma }$) and Comparative Fit Index (CFI) to exceed .95 (Garnier‐Villarreal & Jorgensen, 2020). Based on these criteria, the models demonstrated a good fit.

**FIGURE 1:**
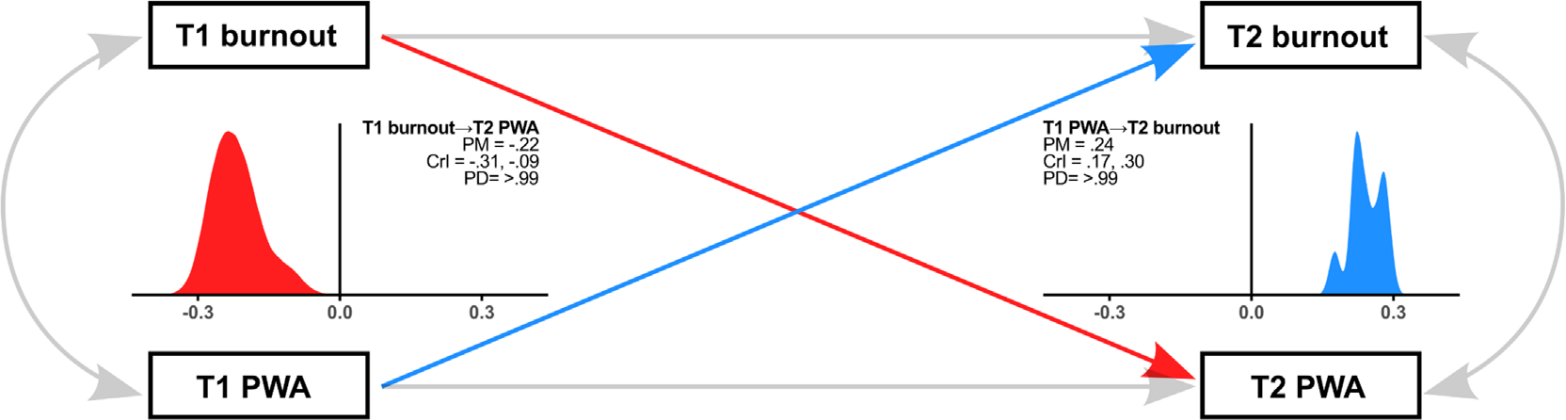
Model scheme*.* Note: PWA – perceived work ability. Red color denotes the cross‐lagged relation of Time 1 (T1) burnout to Time 2 (T2) PWA, with the respective density area of the posterior distribution. Blue color denotes the cross‐lagged relation of Time 1 (T1) PWA to Time 2 (T2) burnout, with the respective density area of the posterior distribution. Made with *ggplot2* (Wickham, 2016).

We estimated two structural equation models. Figure 1 presents the basic structure of both models. The first specification treated burnout and PWA as single latent constructs measured at two time points (T1 and T2). It included (a) autoregressive stability paths from each construct at T1 to itself at T2, (b) two cross‐lagged paths—Burnout_T1 → PWA_T2 and PWA_T1 → Burnout_T2, and (c) within‐time residual covariances between burnout and PWA at T1 and T2. This model directly tested hypotheses that higher burnout predicts lower subsequent PWA among teachers (Hypothesis 1) and that lower PWA predicts higher subsequent burnout (Hypothesis 2). Demographic covariates—gender (binary; men as the reference category), age, and years of practice—were included as predictors of both T2 outcomes to adjust for potential confounding.

As a post hoc, facet‐level specification, we decomposed burnout into the three factors (physical exhaustion, cognitive weariness, emotional exhaustion) and PWA among teachers into the five factors (teaching organization, instructional management, teacher‐staff interaction, navigating difficult situations, non‐teaching responsibilities). The same autoregressive structure was retained, and cross‐lagged paths were estimated from each burnout facet at T1 to each PWA facet at T2 and vice versa, while allowing within‐time residual covariances and including the same covariates for the T2 outcomes. This exploratory model was designed to identify which specific facets account for any whole‐scale associations observed in the initial model.

We did not include any latent variables in our CLPM models because of their high complexity. Instead, we relied on the structural after measurement (SAM) approach (Rosseel & Loh, 2024), which separates the measurement and structural components of the model to reduce computational demands and improve estimation stability. We first fitted a measurement model comprising CFA to estimate the latent variable scores for burnout and PWA. This step ensured that the two latent constructs were adequately captured and that their measurement properties were validated. Then, we used the extracted factor scores as observed variables in our CLPMs. This approach allowed us to focus on the structural relationships between burnout and PWA without the added complexity of estimating the measurement model simultaneously. We standardized all variables before fitting the models.

## RESULTS

For the sake of brevity, we report and interpret only the cross‐lagged relations, which speak directly to our hypotheses and the facet‐level follow‐up. Full parameter estimates are presented in Supplementary Table S4 (simple specification) and Supplementary Table S5 (facet‐level specification).

**FIGURE 2:**
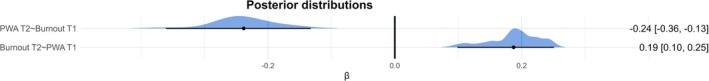
Posterior distributions of cross‐lagged relations in the simple model without the division into the factors*.* Note: PWA – perceived work ability. T1 – Time 1. T2 – Time 2.

In the simple specification (whole scales), the posterior distributions for the two cross‐lagged paths are shown in Figure 2. Consistent with Hypothesis 1, higher burnout at T1 predicted lower PWA among teachers at T2. In contrast to Hypothesis 2, higher PWA at T1 predicted higher burnout at T2. These findings therefore support the anticipated detrimental effect of burnout on subsequent PWA, whereas the reverse directional effect contradicts our prior expectation (see Table 1 for detailed results).

**TABLE 1:** Full model results.

	Imputed model			Non‐imputed model		
	PM	95% CrI	PD	PM	95% CrI	PD
**Cross‐lagged relations**						
T2 PWA ~ T1 burnout (H1)	−.22	[−.31, −.09]	>.99	−.29	[−.37, −.21]	>.99
T2 burnout ~ T1 PWA (H2)	.24	[.17, .30]	>.99	.25	[.23, .28]	>.99
**Stability relations**						
T2 PWA ~ T1 PWA	.48	[.38, .58]	>.99	.50	[.42, .58]	>.99
T2 burnout ~ T1 burnout	1.08	[1.04, 1.12]	>.99	1.11	[1.09, 1.14]	>.99
**Cross‐sectional relations**						
T1 PWA ~ ~ T1 burnout	−.57	[−.66, −.50]	>.99	−.63	[−.74, −.53]	>.99
T2 PWA ~ ~ T2 burnout	−.18	[−.22, −.14]	>.99	−.14	[−.16, −.12]	>.99
**Control effects**						
T2 PWA ~ gender	−.10	[−.26, .07]	.87	−.02	[−.08, .04]	0.72
T2 burnout ~ gender	.02	[−.05, .08]	.72	.01	[−.01, .02]	0.72
T2 PWA ~ age	.00	[−.12, .14]	.58	.01	[−.11, .13]	0.55
T2 burnout ~ age	.01	[−.04, .06]	.59	.00	[−.04, .03]	0.55
T2 PWA ~ years of practice	.09	[−.04, .21]	.92	.10	[−.02, .22]	0.94
T2 burnout ~ years of practice	−.03	[−.09, .01]	.94	−.03	[−.07, .01]	0.94
age ~ ~ years of practice	.88	[.80, .96]	>.99	.86	[.75, .99]	>.99
**Variances**						
T1 PWA	1.00	[.91, 1.10]	>.99	1.00	[.88, 1.14]	>.99
T2 PWA	.58	[.48, .65]	>.99	.46	[.40, .52]	>.99
T1 burnout	1.00	[.91, 1.10]	>.99	1.00	[.88, 1.14]	>.99
T2 burnout	.07	[.05, .09]	>.99	.04	[.04, .05]	>.99
age	1.00	[.91, 1.10]	>.99	1.00	[.88, 1.14]	>.99
years of practice	1.00	[.91, 1.10]	>.99	1.00	[.88, 1.14]	>.99

*Note*: PWA – perceived work ability. T1 – Time 1, T2 – Time 2. H1 – Hypothesis 1, H2 – Hypothesis 2.

**FIGURE 3:**
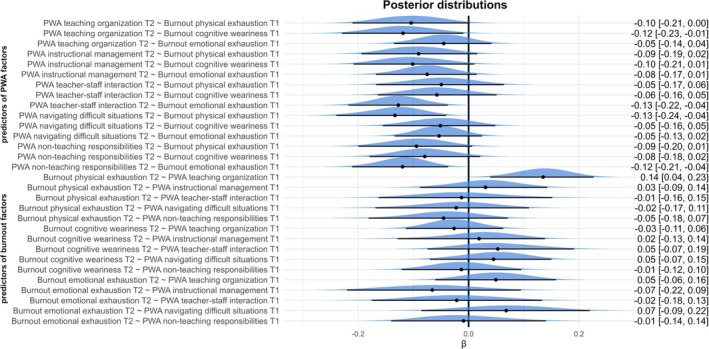
Posterior distributions of cross‐lagged relations in the complex model with the division into the factors. Note: PWA – perceived work ability. T1 – Time 1, T2 – Time 2.

In the facet‐level specification (three burnout facets × five PWA facets), Figure 3 summarizes the cross‐lagged paths between facets. A consistent pattern emerged in the burnout → PWA direction: physical exhaustion at T1 predicted lower PWA in teaching organization, navigating difficult situations, and managing non‐teaching responsibilities at T2; cognitive weariness at T1 predicted lower PWA in teaching organization and instructional management at T2; and emotional exhaustion at T1 predicted lower PWA in teacher‐staff interaction as well as in non‐teaching responsibilities at T2. In the PWA → burnout direction, only one credible effect was observed: higher PWA in teaching organization at T1 predicted higher physical exhaustion at T2.

Taking together, these results suggest that the whole‐scale association between burnout and lower PWA is distributed across several theoretically coherent facet‐level links rather than being driven by a single domain, whereas the PWA‐to‐higher burnout pattern appears to be localized to the link between PWA in teaching organizations and subsequent physical exhaustion.

## DISCUSSION

In addressing the gaps and limitations in previous research, our study investigated the bidirectional relationship between burnout and PWA among primary and lower secondary school teachers using Bayesian cross‐lagged panel modeling (CLPM). While inspired by the work of Viotti et al. (2019), the present study is not a replication; rather, it applies a different theoretical framework, employs a distinct methodological approach, and focuses on a different educational context. By applying the JD‐R model (Bakker et al., 2005, 2007; Bakker & Demerouti, 2007, 2017; Demerouti & Bakker, 2011) and the COR theory (Hobfoll, 1989, 2001), we aimed to establish a comprehensive framework for understanding the dynamic interplay between these constructs. Nevertheless, the conceptual, contextual, and methodological differences between the two studies limit the potential for direct comparability. Accordingly, the present research should be viewed as a complementary contribution that enriches the literature by examining the burnout–PWA relationship in a different educational context using a profession‐specific measurement instrument.

Our study highlighted the effect of burnout on PWA over time. The findings of the present study are consistent with the expectations outlined in the JD‐R model (Schaufeli, 2017), which posits that burnout contributes to health deterioration and other negative work‐related outcomes. Furthermore, they align with the results of several cross‐sectional studies among various helping professional groups, including teachers (e.g., Debets et al., 2022; Hakanen et al., 2006; Hatch et al., 2018; Hlaďo et al., 2020; Pranjic & Bilic, 2014; Seibt et al., 2009, for example). However, our findings contradict the study by Viotti et al. (2019), which also used a cross‐lagged design and found no evidence of any burnout dimension (i.e., exhaustion, enthusiasm, and cynicism) affecting PWA among early childhood educators. The authors explain their finding by suggesting that chronic levels of burnout may require a prolonged period to deplete an individual's functional capacity to work. The discrepancy between the findings of Viotti et al. (2019) and our results may be attributable not only to the elevated demands and risks inherent in primary and lower secondary teaching but also to differences in measurement instruments. Whereas Viotti et al. (2019) assessed burnout using the Spanish Burnout Inventory (Guidetti et al., 2018), which captures psychological exhaustion, enthusiasm toward the job, and cynicism, our study employed the Shirom–Melamed Burnout Inventory (Ptáček et al., 2017; Shirom & Melamed, 2006), which focuses on the depletion of physical, emotional, and cognitive energy resources. The process of energy depletion, manifested as physical fatigue, emotional exhaustion, and cognitive weariness, can significantly impair primary and lower secondary teachers' PWA, even over a relatively short period. This decline may become so pronounced that, from the teachers' subjective perspective, they perceive themselves as unable to meet job demands or to fulfill their responsibilities effectively. Such deterioration is particularly concerning given the excessive job demands routinely faced in primary and lower secondary education, which require a high level of PWA to manage effectively.

Post hoc facet‐level analyses revealed distinct patterns linking specific dimensions of burnout to particular facets of PWA. In the burnout → PWA direction, physical exhaustion predicted subsequent declines in PWA in teaching organization, navigating difficult situations, and non‐teaching responsibilities one year later. These findings suggest that a loss of physical energy may undermine a teacher's capacity to effectively organize instruction, manage diverse student groups, and respond efficiently to demanding interpersonal or conflict situations involving students and their parents. Moreover, it may result in a diminished capacity to fulfill work‐related demands such as administrative tasks, student supervision, and participation in school and extracurricular events. Cognitive fatigue predicted a lower PWA in teaching organization and instructional management. Teachers experiencing cognitive fatigue thus exhibit a reduced capacity to perform tasks related to the development of students' knowledge and skills and to the provision of feedback—posing a potential risk for lower student academic achievement. Emotional exhaustion emerged as a predictor of lower PWA in teacher‐staff interaction and non‐teaching responsibilities. This suggests that emotional depletion can impair teachers' capacity to actively engage in teamwork as well as in non‐instructional duties. This finding underscores the comprehensive nature of burnout's impact on PWA. Overall, these findings underscore the multifaceted impact of burnout on PWA. In practical terms, they indicate that interventions designed to preserve or enhance PWA should address all dimensions of burnout simultaneously, as declines in physical, emotional, or cognitive energy are likely to compromise overall work functioning across the full spectrum of professional responsibilities.

One of the most unanticipated findings of our study is that teachers with higher PWA reported increased levels of burnout, whereas those with lower PWA experienced lower levels of burnout over time. This result stands in contrast to previous research (Converso et al., 2021; Viotti et al., 2017) and the COR theory (Hobfoll, 1989, 2001; Hobfoll & Shirom, 2001). To further clarify this unexpected finding, it is essential to distinguish PWA from related constructs such as general and job self‐efficacy, work engagement, and job satisfaction. Self‐efficacy refers to an individual's belief in their ability to successfully perform specific tasks, while job self‐efficacy pertains to perceived competence in performing one's job effectively. In contrast, PWA represents a broader, capacity‐based self‐assessment of one's ability to continue working under current conditions (Brady et al., 2020; Cadiz et al., 2019; McGonagle et al., 2015). Similarly, work engagement is typically conceptualized as an affective‐motivational state characterized by vigor, dedication, and absorption (Schaufeli et al., 2006). PWA, however, does not reflect emotional involvement or enthusiasm for one's work, but rather a more functional and capacity‐focused appraisal of one's ability to cope with job demands. In contrast, job satisfaction reflects an evaluative appraisal of job conditions and the work environment, not a judgment of one's capacity to work (Judge et al., 2001).

Whereas constructs such as self‐efficacy and work engagement are generally associated with motivational resources and positive affect (Bandura, 1997; Schaufeli et al., 2006), PWA may instead indicate an individual's capacity to endure demanding or adverse conditions. In professions such as teaching, individuals with high PWA may be viewed as able to manage greater workloads, potentially resulting in prolonged exposure to stressors (Day & Gu, 2010). Consequently, high PWA may not always act as a buffer against strain, but as an indicator of greater tolerance for it, potentially increasing the risk of burnout (McGonagle et al., 2022b).

As a theoretical foundation for further understanding of the observed association between PWA and burnout, the concept of self‐endangering work behavior (Dettmers et al., 2016) and the effort–reward imbalance model (Siegrist, 2017) offer particularly promising frameworks. Considering the concept of self‐endangering work behavior, teachers with high PWA may be especially vulnerable to self‐endangering strategies such as working through exhaustion, postponing recovery, or consistently overextending themselves. These behaviors are largely driven by a strong internalized sense of functional capacity to meet job demands and can be understood as coping responses to high workloads and persistent occupational pressures. Although such strategies may be effective in achieving short‐term work goals, they are dysfunctional for occupational health, as they undermine psychological resilience and increase susceptibility to burnout (Dettmers et al., 2016). The effort–reward imbalance model provides a complementary perspective by highlighting the detrimental effects of sustained effort that is not matched by adequate rewards such as recognition, autonomy, or recovery opportunities. It also emphasizes overcommitment, understood as a personal tendency toward excessive work‐related striving, a key factor that may intensify this imbalance. High PWA may therefore increase burnout risk indirectly by fostering both self‐endangering behaviors and overcommitment in response to chronic job demands.

To further explore this phenomenon, we conducted a post hoc analysis to investigate the effects of individual PWA dimensions on teacher burnout. The results indicated that only PWA in a teaching organization was significantly associated with physical burnout. This dimension reflects the capacity to organize individual and group work, differentiate instruction to address diverse student needs, and manage heterogeneous classrooms. A high level of such capacity may result in sustained overextension and the depletion of physical resources, thereby increasing the risk of physical burnout. Teachers who perceive themselves as highly capable in these domains are likely to engage more frequently in physically demanding instructional activities, such as moving continuously around the classroom, interacting with multiple student groups, and managing diverse instructional materials. Unlike purely cognitive or administrative demands, these pedagogical tasks impose direct and cumulative strain on the body, contributing to somatic wear and diminishing opportunities for micro‐recovery during lessons.

From a theoretical perspective, the findings of our study align with the concept of self‐endangering work behavior (Dettmers et al., 2016) and the effort–recovery model (Meijman & Mulder, 1998). Teachers with high PWA in teaching organizations may be more prone to adopting self‐endangering strategies such as prolonged overextension, postponing necessary breaks, or persistently striving to meet diverse student needs despite insufficient recovery opportunities. The effort–recovery model provides a complementary explanation, positing that repeated physical exertion without adequate rest fosters cumulative fatigue that progressively undermines physical resilience and heightens susceptibility to physical burnout. Taken together, the findings of our study indicate that a capacity in teaching organization, commonly regarded as a valuable professional asset, may, under sustained and demanding conditions, function as a risk factor by contributing to physical strain and burnout.

### Theoretical implications

The present study offers several important theoretical implications. First, it provides empirical support for the JD‐R model by demonstrating that PWA functions as a work‐related outcome negatively affected by burnout (Schaufeli et al., 2006). The observed temporal association further reinforces the health impairment process within the JD‐R framework, suggesting that burnout resulting from chronically elevated job demands in the teaching profession can, over time, lead to a decline in teachers' occupational functioning. This temporal link between burnout and the subsequent decreases in PWA expands the JD‐R model's nomological network of outcomes beyond traditional indicators such as health, sickness absence, poor performance, and disengagement (Schaufeli & Taris, 2014). These findings underscore the theoretical and practical relevance of incorporating PWA into future empirical investigations addressing the long‐term consequences of chronic job stress. Moreover, they highlight the need for theoretical models that account for time‐lagged changes resulting from psychological strain.

Second, the finding that higher levels of PWA can contribute to the development of burnout reinforces the view of PWA as a dynamic, context‐dependent indicator of occupational health (Brady et al., 2020), rather than personal resources. This interpretation is further reinforced by our additional finding that PWA does not exert a protective effect on burnout levels. Our results thus provide empirical support for the argument that PWA is misaligned with the conceptual criteria typically used to define personal resources within the JD‐R model (Xanthopoulou et al., 2009).

Furthermore, the present study challenges the underlying assumptions of the COR theory (Hobfoll, 1989, 2001) by demonstrating that high levels of PWA may not serve a protective function, but rather may signal increased exposure to job demands, which can accelerate burnout. These findings call for a reconceptualization of PWA within existing theoretical frameworks, as our data suggest that it functions neither as a personal resource nor as a health‐related resource (Airila et al., 2014; Viotti et al., 2019).

Finally, while the JD‐R model and the COR theory each offer valuable insights, our findings suggest that neither is sufficient on its own to fully capture the complex dynamics between burnout and PWA. The risk‐enhancing role of high PWA underscores the need for a more integrative theoretical framework that accounts for the strain‐amplifying potential of PWA under conditions of chronic job demands.

### Practical implications

The findings from the present study have relevant practical implications. Our results have highlighted the centrality of supporting burnout in preventing WA depletion among teachers. Drawing on the JD‐R model (Bakker & Demerouti, 2007) and previous research on the antecedents of teacher burnout (Hlado et al., 2025a; Menon et al., 2024), schools can mitigate the risk of WA depletion by enhancing job resources. This includes promoting teacher autonomy, ensuring adequate support from supervisors and colleagues, fostering collaboration and mentoring opportunities, providing constructive feedback and recognition, and offering systematic professional development. Reducing excessive job demands and optimizing workload allocation based on individual teacher needs (Hlado & Harvankova, 2024) can also help preserve adequate time for rest, maintain a healthy work‐life balance, and prevent the depletion of capacity to perform job requirements.

Alongside these general strategies, structured and evidence‐based intervention frameworks can also be recommended as effective means of addressing the issue. For example, Ahmadi et al. (2023) proposed a classification of motivational and need‐supportive behaviors that can inform teacher‐centered burnout prevention efforts. This structured approach to fostering psychological need satisfaction can inform targeted training for school leaders, emphasizing autonomy‐supportive communication, recognition of teacher efforts, and sensitivity to individual needs. Teachers' ability to manage stressful situations, and thereby prevent burnout, can be further enhanced through interventions aimed at building psychological resilience or through mindfulness‐based programs (Hidajat et al., 2023b). One such example is the Stress Prevention for Teachers (PREST) program (Unterbrink et al., 2012), which combines psychoeducation, cognitive‐behavioral techniques, and relaxation training. This program has been shown to significantly reduce emotional exhaustion among teachers. Similarly, the Cultivating Awareness and Resilience in Education (CARE) program (Jennings et al., 2019), a mindfulness‐based professional development initiative, has demonstrated positive effects on reducing teacher stress and improving emotional regulation.

Our study further suggests that high PWA may present a risk factor for teachers, as individuals with a greater capacity to meet job demands may be more vulnerable to workload overload. The foundation for prevention lies in the finding that teachers lack a clear understanding of PWA (Hlado & Harvankova, 2024). This qualitative study revealed that teachers view physical and mental health, along with professional competencies, as the core components of PWA. They are unfamiliar with conceptualizing PWA as a balance between personal resources and job demands. This may lead teachers to insufficiently recognize that while high PWA allows them to handle a large workload, they must be cautious not to deplete this resource. As the COR theory (Hobfoll, 2001) indicates, the loss of resources initiates a cycle of further depletion, which can result in work‐related stress and burnout. We therefore recommend, in line with the study by Hlado and Harvankova (2024), supporting an informational process aimed at increasing awareness of the PWA construct and strategies for its maintenance. This will enable teachers to take responsibility for their PWA, thereby preventing burnout.

At the policy level, our findings highlight the need for systemic reforms to support teacher well‐being and sustain PWA. Based on OECD (2018, 2020) insights, we recommend limiting excessive teaching hours, reducing administrative burdens, and adjusting student–teacher ratios. In this regard, post hoc analysis suggests that the systematic integration of teaching assistants into heterogeneous classrooms comprising students with diverse needs may serve as an effective preventive measure against physical burnout, since their presence can substantially facilitate classroom management and reduce teachers' workload (Skipp & Hopwood, 2019). Initiatives such as the Workload Challenge (OECD, 2020) illustrate that well‐designed workload reduction strategies can simultaneously improve professional efficiency and mitigate stress among teachers, while maintaining high standards of educational quality. National professional development frameworks at both the pre‐service and in‐service levels should explicitly address teacher well‐being, resilience, and burnout prevention (Day & Gu, 2013). Furthermore, integrating well‐being indicators into teacher standards and school evaluation frameworks can institutionalize psychosocial risk prevention and foster shared responsibility for maintaining teachers' PWA and long‐term professional sustainability (OECD, 2018).

### Limitations and future research

Our study has several limitations that should be considered when interpreting the findings. First, while the Bayesian CLPM offers valuable insights into the bidirectional relationships between variables, it cannot establish definitive causal claims. Additionally, the CLPM framework does not disentangle within‐person (state‐like) and between‐person (trait‐like) variance, potentially conflating dynamic changes with stable individual differences (Lucas, 2023). Despite these limitations, given the constraints of our data, which included only two waves, we believe that the Bayesian CLPM represents the most suitable approach to address our hypotheses.

Second, variables under study were assessed via self‐reports, reflecting teachers' subjective evaluations. While self‐report instruments are well‐established tools for capturing internal experiences, they are susceptible to potential biases, such as social desirability and common method variance. These limitations are particularly pronounced when addressing personally or professionally sensitive constructs. Moreover, self‐assessments may not fully align with objective indicators or external evaluations. Future research would benefit from incorporating multi‐method approaches, including physiological stress markers, behavioral indicators of work performance, and external assessments from supervisors or co‐workers. Such triangulation would offer a more comprehensive understanding of burnout and PWA and strengthen the robustness and validity of findings.

Third, our sample is non‐representative. The study was conducted with a relatively homogeneous sample of primary and lower secondary school teachers from the Czech Republic, which constrains the generalizability of the findings to other populations, such as preschool, upper secondary school, and university‐level educators, as well as teaching assistants, headteachers, or professionals from different cultural and occupational contexts. In particular, certain cultural characteristics of the Czech educational system, such as its centralized curriculum, the relative autonomy of individual schools, and appreciations of teachers (Pešková et al., 2019; Tomsikova et al., 2020), may influence both burnout and PWA in ways that differ from educational contexts in other countries. These contextual factors may moderate the psychological processes under investigation and thus may constrain the applicability of our findings in other international settings. Additionally, the sample is characterized by a significant proportion of female participants, reflecting the gender structure of the Czech teaching workforce (MEYS, 2024). This demographic pattern is not unique to the Czech Republic; similar gender distributions are observed across other OECD countries (OECD, 2021). However, the gender composition may have affected the overall level of burnout and PWA, as prior research suggests that women tend to report lower WA and higher levels of emotional exhaustion (Airila et al., 2014; Hlaďo et al., 2017). These gender‐specific characteristics may have influenced the observed patterns and should be taken into account when interpreting the findings. Given the stated limitations, the present study does not aim to estimate prevalence rates but rather to examine relationships that may inform future research and practice. To strengthen the validity and generalizability of the identified associations, future research should employ internationally comparative samples and investigate how sociocultural and institutional factors may influence the relationship between burnout and PWA. Moreover, considering the persistent gender imbalance in the teaching profession and existing evidence of gender‐related differences in both burnout and PWA, future studies should conduct gender‐specific analyses to develop a more nuanced and differentiated understanding of these psychological processes.

Fourth, due to the complexity of the model relative to the sample size, we were unable to perform mediation analyses that could have helped clarify the counterintuitive findings observed in our study. We therefore recommend that future research with larger samples investigate potential mediating mechanisms to better understand the relationship between PWA and burnout. These mechanisms may include job‐related stressors, such as job demands, work overload, effort–reward imbalance, and overcommitment, as well as job resources, including supervisor support and collegial support. Future research should also aim to extend the theoretical scope of the model. Multi‐group structural equation modeling or multilevel approaches could be employed to examine whether PWA varies across different teaching levels, degrees of teaching experience (e.g., novice vs. veteran teachers), or national contexts, and to explore the ways in which such differences manifest. Such approaches could help determine whether the strength or direction of associations between PWA and burnout differs across these groups.

Lastly, our inability to incorporate the multilevel structure of the data due to the high complexity of the models compared to the sample size—teachers nested within schools—is another limitation. A multilevel modeling approach could provide a more nuanced understanding of how school‐level factors or contextual influences interact with the relationships we examined. Future research should consider employing hierarchical linear modeling (HLM) or other multilevel techniques to better capture the nested nature of educational data. Accounting for both individual‐ and school‐level variance would enable researchers to disentangle personal and contextual influences on burnout and PWA, and may uncover school‐related factors (e.g., organizational support, leadership practices) that moderate these dynamics. Incorporating such context‐sensitive designs would open new avenues for targeted interventions at both the individual and organizational levels.

## CONCLUSIONS

The present study investigated the bidirectional relationship between burnout and PWA among primary and lower secondary school teachers, utilizing a two‐wave study design and cross‐lagged panel modeling (CLPM). The results indicated that burnout is associated with a decrease in PWA over time. Unexpectedly, higher PWA was found to be linked with higher burnout over time. These findings suggest that, rather than acting as a protective factor, higher PWA may, in some cases, intensify burnout. From a theoretical standpoint, it is possible to explain this finding by suggesting that teachers with a greater capacity to meet job demands tend to assume additional responsibilities, which may, in turn, increase their stress levels and ultimately lead to burnout. The present study has important practical implications, highlighting the need for burnout prevention strategies to protect against PWA depletion. Increasing awareness of the PWA construct and strategies for its maintenance may contribute to the prevention of burnout and support the sustained preservation of PWA throughout the course of one's professional life.

## AUTHOR CONTRIBUTIONS

Conceptualization: P.H.; methodology: P.H., T.L., L.J.; data collection: P.H., L.J., K.H.; formal analysis: T.L.; funding acquisition: P.H.; writing – original draft: P.H., T.L.; writing – review and editing: P.H., T.L. All the authors have read and agreed to the published version of the manuscript.

## ETHICS STATEMENT

All procedures in this study involving human participants were performed in accordance with the ethical standards of the institutional research committee. Approval for the study was obtained from the Research Ethics Committee of Masaryk University under reference number EKV‐2022‐031 prior to data collection.

## CONFLICT OF INTEREST STATEMENT

The authors declare no competing interests.

## INFORMED CONSENT

Informed consent was given by all participants. Teachers were given the option to contact the research team before, during, and after the data collection and the option to withdraw their consent and stop participating in the research at any time.

## Supporting information


**Table S1.** Non‐imputed model results – without factors.
**Table S2**. Non‐imputed model results – with factors.
**Table S3**. Fit indices.
**Table S4**. Imputed model results – without factors.
**Table S5**. Imputed model results – with factors.

## Data Availability

The datasets generated during and analyzed during the current study and supplementary material are available from: 10.7910/DVN/8RGHK6
