# Supplementary material for: Bidirectional relationship between burnout and perceived work ability: Evidence from a two‐wave study among teachers
Source: Appl Psychol Health Well Being. 2025 Sep 9;17(5):e70075. doi: 10.1111/aphw.70075 (PMC12418066; doi:10.1111/aphw.70075)
Supplement: Supplementary file 1 — Table S1. Non‐imputed model results – without factors. Table S2. Non‐imputed model results – with factors. Table S3. Fit indices. Table S4. Imputed model results – without factors. Table S5. Imputed model results – with factors. [file APHW-17-0-s001.pdf]

Supplementary Material for
Bidirectional Relationship Between Burnout and Perceived Work Ability: Evidence from a Two-Wave Study among Teachers

Supplementary Table S1. Non-imputed model results – without factors.

Effect name	M	CI_lower	CI_upper
**Cross-lagged relations**			
PWA (T2) ~ Burnout (T1)	−0.28	−0.36	−0.20
Burnout (T2) ~ PWA (T1)	0.25	0.23	0.28
**Autoregressive effects**			
PWA (T2) ~ PWA (T1)	0.46	0.38	0.55
Burnout (T2) ~ Burnout (T1)	1.11	1.09	1.14
**Control effects**			
Burnout (T1) ~ Age	−0.09	−0.26	0.09
Burnout (T1) ~ Gender	0.06	−0.03	0.15
Burnout (T1) ~ Years of prctc	−0.04	−0.22	0.13
Burnout (T2) ~ Age	0.00	−0.04	0.04
Burnout (T2) ~ Gender	0.01	−0.01	0.02
Burnout (T2) ~ Years of prctc	−0.04	−0.08	0.00
PWA (T1) ~ Age	0.11	−0.07	0.29
PWA (T1) ~ Gender	−0.01	−0.10	0.08
PWA (T1) ~ Years of prctc	0.07	−0.10	0.25
PWA (T2) ~ Age	−0.01	−0.14	0.12
PWA (T2) ~ Gender	−0.02	−0.08	0.05
PWA (T2) ~ Years of prctc	0.13	0.00	0.26
**Covariances**			
Age ~~ Years of prctc	0.86	0.76	0.99
Burnout (T1) ~~ PWA (T1)	−0.60	−0.72	−0.51
Burnout (T2) ~~ PWA (T2)	−0.15	−0.17	−0.13
Gender ~~ Age	0.13	0.04	0.22
Gender ~~ Years of prctc	0.15	0.06	0.24
**Intercepts**			
Intercept: Age	0.00	−0.09	0.09
Intercept: Burnout (T1)	0.00	−0.09	0.09
Intercept: Burnout (T2)	0.00	−0.02	0.02
Intercept: Gender	0.00	−0.09	0.09
Intercept: PWA (T1)	0.00	−0.09	0.09
Intercept: PWA (T2)	0.00	−0.06	0.06
Intercept: Years of prctc	0.00	−0.09	0.09
**Variances**			
Variance: Age	1.00	0.89	1.14
Variance: Burnout (T1)	1.00	0.88	1.13
Variance: Burnout (T2)	0.04	0.04	0.05
Variance: Gender	1.01	0.89	1.14
Variance: PWA (T1)	0.98	0.86	1.12
Variance: PWA (T2)	0.51	0.45	0.58
Variance: Years of practice	1.00	0.89	1.14

Supplementary Table S2. Non-imputed model results – with factors.

Effect name	M	CI_lower	CI_upper
**Cross-lagged relations**			
Burnout: cognitive weariness (T2) ~ PWA: instructional management (T1)	−0.02	−0.13	0.09
Burnout: cognitive weariness (T2) ~ PWA: navigating difficult situations (T1)	0.07	−0.05	0.19
Burnout: cognitive weariness (T2) ~ PWA: non-teaching responsibilities (T1)	−0.04	−0.16	0.08
Burnout: cognitive weariness (T2) ~ PWA: teacher-staff interaction (T1)	0.07	−0.07	0.20
Burnout: cognitive weariness (T2) ~ PWA: teaching organization (T1)	0.00	−0.10	0.10
Burnout: emotional exhaustion (T2) ~ PWA: instructional management (T1)	−0.10	−0.23	0.03
Burnout: emotional exhaustion (T2) ~ PWA: navigating difficult situations (T1)	0.09	−0.05	0.23
Burnout: emotional exhaustion (T2) ~ PWA: non-teaching responsibilities (T1)	−0.05	−0.19	0.09
Burnout: emotional exhaustion (T2) ~ PWA: teacher-staff interaction (T1)	0.02	−0.14	0.19
Burnout: emotional exhaustion (T2) ~ PWA: teaching organization (T1)	0.06	−0.06	0.18
Burnout: physical exhaustion (T2) ~ PWA: instructional management (T1)	0.01	−0.10	0.13
Burnout: physical exhaustion (T2) ~ PWA: navigating difficult situations (T1)	−0.01	−0.14	0.12
Burnout: physical exhaustion (T2) ~ PWA: non-teaching responsibilities (T1)	−0.11	−0.23	0.02
Burnout: physical exhaustion (T2) ~ PWA: teacher-staff interaction (T1)	0.03	−0.11	0.18
Burnout: physical exhaustion (T2) ~ PWA: teaching organization (T1)	0.14	0.03	0.24
PWA: teaching organization (T2) ~ Burnout: cognitive weariness (T1)	−0.12	−0.22	−0.02
PWA: teaching organization (T2) ~ Burnout: emotional exhaustion (T1)	−0.07	−0.16	0.03
PWA: teaching organization (T2) ~ Burnout: physical exhaustion (T1)	−0.10	−0.20	0.00
PWA: instructional management (T2) ~ Burnout: cognitive weariness (T1)	−0.11	−0.21	0.00
PWA: instructional management (T2) ~ Burnout: emotional exhaustion (T1)	−0.09	−0.19	0.00
PWA: instructional management (T2) ~ Burnout: physical exhaustion (T1)	−0.09	−0.19	0.01
PWA: teacher-staff interaction (T2) ~ Burnout: cognitive weariness (T1)	−0.06	−0.16	0.04
PWA: teacher-staff interaction (T2) ~ Burnout: emotional exhaustion (T1)	−0.14	−0.23	−0.05
PWA: teacher-staff interaction (T2) ~ Burnout: physical exhaustion (T1)	−0.05	−0.15	0.05
PWA: navigating difficult situations (T2) ~ Burnout: cognitive weariness (T1)	−0.05	−0.15	0.04
PWA: navigating difficult situations (T2) ~ Burnout: emotional exhaustion (T1)	−0.07	−0.16	0.01
PWA: navigating difficult situations (T2) ~ Burnout: physical exhaustion (T1)	−0.13	−0.23	−0.04
PWA: non-teaching responsibilities (T2) ~ Burnout: cognitive weariness (T1)	−0.08	−0.18	0.02
PWA: non-teaching responsibilities (T2) ~ Burnout: emotional exhaustion (T1)	−0.15	−0.24	−0.06
PWA: non-teaching responsibilities (T2) ~ Burnout: physical exhaustion (T1)	−0.08	−0.18	0.02
**Autoregressive effects**			
Burnout: cognitive weariness (T2) ~ Burnout: cognitive weariness (T1)	0.72	0.66	0.78
Burnout: emotional exhaustion (T2) ~ Burnout: emotional exhaustion (T1)	0.55	0.48	0.63
Burnout: physical exhaustion (T2) ~ Burnout: physical exhaustion (T1)	0.64	0.57	0.71
PWA: instructional management (T2) ~ PWA: instructional management (T1)	0.23	0.19	0.28
PWA: navigating difficult situations (T2) ~ PWA: navigating difficult situations (T1)	0.33	0.28	0.37
PWA: non-teaching responsibilities (T2) ~ PWA: non-teaching responsibilities (T1)	0.26	0.22	0.29
PWA: teacher-staff interaction (T2) ~ PWA: teacher-staff interaction (T1)	0.27	0.22	0.32
**Control effects**			
Burnout: cognitive weariness (T1) ~ Age	−0.08	−0.25	0.10
Burnout: cognitive weariness (T1) ~ Gender	0.10	0.01	0.19
Burnout: cognitive weariness (T1) ~ Years of practice	−0.01	−0.19	0.17
Burnout: cognitive weariness (T2) ~ Age	0.00	−0.12	0.12
Burnout: cognitive weariness (T2) ~ Gender	−0.01	−0.07	0.05

Burnout: cognitive weariness (T2) ~ Years of practice	−0.06	−0.18	0.06
Burnout: emotional exhaustion (T1) ~ Age	−0.02	−0.20	0.16
Burnout: emotional exhaustion (T1) ~ Gender	−0.03	−0.12	0.06
Burnout: emotional exhaustion (T1) ~ Years of practice	−0.06	−0.24	0.12
Burnout: emotional exhaustion (T2) ~ Age	0.04	−0.10	0.18
Burnout: emotional exhaustion (T2) ~ Gender	0.01	−0.07	0.08
Burnout: emotional exhaustion (T2) ~ Years of practice	−0.10	−0.24	0.05
Burnout: physical exhaustion (T1) ~ Age	−0.09	−0.27	0.09
Burnout: physical exhaustion (T1) ~ Gender	0.05	−0.04	0.14
Burnout: physical exhaustion (T1) ~ Years of practice	0.01	−0.17	0.19
Burnout: physical exhaustion (T2) ~ Age	−0.08	−0.20	0.05
Burnout: physical exhaustion (T2) ~ Gender	0.07	0.01	0.14
Burnout: physical exhaustion (T2) ~ Years of practice	−0.02	−0.14	0.11
PWA: instructional management (T1) ~ Age	0.05	−0.12	0.23
PWA: instructional management (T1) ~ Gender	0.02	−0.07	0.11
PWA: instructional management (T1) ~ Years of practice	0.09	−0.09	0.26
PWA: instructional management (T2) ~ Age	0.01	−0.13	0.16
PWA: instructional management (T2) ~ Gender	−0.03	−0.10	0.05
PWA: instructional management (T2) ~ Years of practice	0.14	0.00	0.29
PWA: navigating difficult situations (T1) ~ Age	0.12	−0.05	0.29
PWA: navigating difficult situations (T1) ~ Gender	0.01	−0.08	0.10
PWA: navigating difficult situations (T1) ~ Years of practice	0.07	−0.10	0.24
PWA: navigating difficult situations (T2) ~ Age	0.01	−0.13	0.15
PWA: navigating difficult situations (T2) ~ Gender	−0.03	−0.10	0.05
PWA: navigating difficult situations (T2) ~ Years of practice	0.11	−0.02	0.25
PWA: non-teaching responsibilities (T1) ~ Age	0.08	−0.10	0.25
PWA: non-teaching responsibilities (T1) ~ Gender	−0.04	−0.12	0.05
PWA: non-teaching responsibilities (T1) ~ Years of practice	0.01	−0.16	0.18
PWA: non-teaching responsibilities (T2) ~ Age	0.02	−0.12	0.16
PWA: non-teaching responsibilities (T2) ~ Gender	−0.02	−0.09	0.05
PWA: non-teaching responsibilities (T2) ~ Years of practice	0.17	0.03	0.31
PWA: teacher-staff interaction (T1) ~ Age	0.10	−0.07	0.28
PWA: teacher-staff interaction (T1) ~ Gender	−0.03	−0.12	0.06
PWA: teacher-staff interaction (T1) ~ Years of practice	0.10	−0.07	0.27
PWA: teacher-staff interaction (T2) ~ Age	0.03	−0.11	0.18
PWA: teacher-staff interaction (T2) ~ Gender	−0.02	−0.10	0.05
PWA: teacher-staff interaction (T2) ~ Years of practice	0.16	0.02	0.30
PWA: teaching organization (T1) ~ Age	0.13	−0.04	0.31
PWA: teaching organization (T1) ~ Gender	0.03	−0.06	0.12
PWA: teaching organization (T1) ~ Years of practice	0.01	−0.17	0.18
PWA: teaching organization (T2) ~ Age	0.02	−0.13	0.16
PWA: teaching organization (T2) ~ Gender	−0.03	−0.11	0.04
PWA: teaching organization (T2) ~ PWA: teaching organization (T1)	0.24	0.20	0.28
PWA: teaching organization (T2) ~ Years of practice	0.15	0.00	0.29
**Covariances**			
Age ~~ Years of practice	0.87	0.75	0.99
Burnout: cognitive weariness (T1) ~~ Burnout: emotional exhaustion (T1)	0.58	0.49	0.69
Burnout: cognitive weariness (T1) ~~ PWA: instructional management (T1)	−0.39	−0.48	−0.30

Burnout: cognitive weariness (T1) ~~ PWA: navigating difficult situations (T1)	−0.38	−0.47	−0.29
Burnout: cognitive weariness (T1) ~~ PWA: non-teaching responsibilities (T1)	−0.41	−0.50	−0.32
Burnout: cognitive weariness (T1) ~~ PWA: teacher-staff interaction (T1)	−0.38	−0.47	−0.29
Burnout: cognitive weariness (T1) ~~ PWA: teaching organization (T1)	−0.37	−0.46	−0.28
Burnout: cognitive weariness (T2) ~~ Burnout emotional exhaustion T2	0.24	0.19	0.30
Burnout: cognitive weariness (T2) ~~ PWA: instructional management (T2)	−0.13	−0.18	−0.08
Burnout: cognitive weariness (T2) ~~ PWA: navigating difficult situations (T2)	−0.13	−0.18	−0.09
Burnout: cognitive weariness (T2) ~~ PWA: non-teaching responsibilities (T2)	−0.13	−0.18	−0.08
Burnout: cognitive weariness (T2) ~~ PWA teacher-staff interaction T2	−0.13	−0.18	−0.08
Burnout: cognitive weariness (T2) ~~ PWA: teaching organization (T2)	−0.15	−0.20	−0.09
Burnout: emotional exhaustion (T1) ~~ PWA: instructional management (T1)	−0.36	−0.46	−0.28
Burnout: emotional exhaustion (T1) ~~ PWA: navigating difficult situations (T1)	−0.39	−0.49	−0.30
Burnout: emotional exhaustion (T1) ~~ PWA: non-teaching responsibilities (T1)	−0.40	−0.50	−0.32
Burnout: emotional exhaustion (T1) ~~ PWA: teacher-staff interaction (T1)	−0.41	−0.50	−0.32
Burnout: emotional exhaustion (T1) ~~ PWA: teaching organization (T1)	−0.38	−0.47	−0.29
Burnout: emotional exhaustion (T2) ~~ PWA: instructional management (T2)	−0.16	−0.22	−0.10
Burnout: emotional exhaustion (T2) ~~ PWA: navigating difficult situations (T2)	−0.14	−0.21	−0.09
Burnout: emotional exhaustion (T2) ~~ PWA: non-teaching responsibilities (T2)	−0.15	−0.22	−0.09
Burnout: emotional exhaustion (T2) ~~ PWA teacher-staff interaction T2	−0.16	−0.22	−0.10
Burnout: emotional exhaustion (T2) ~~ PWA: teaching organization (T2)	−0.17	−0.23	−0.11
Burnout: physical exhaustion (T1) ~~ Burnout: cognitive weariness (T1)	0.64	0.54	0.75
Burnout: physical exhaustion (T1) ~~ Burnout: emotional exhaustion (T1)	0.56	0.46	0.66
Burnout: physical exhaustion (T1) ~~ PWA: instructional management (T1)	−0.31	−0.40	−0.22
Burnout: physical exhaustion (T1) ~~ PWA: navigating difficult situations (T1)	−0.38	−0.48	−0.30
Burnout: physical exhaustion (T1) ~~ PWA: non-teaching responsibilities (T1)	−0.38	−0.47	−0.29
Burnout: physical exhaustion (T1) ~~ PWA: teacher-staff interaction (T1)	−0.34	−0.44	−0.26
Burnout: physical exhaustion (T1) ~~ PWA: teaching organization (T1)	−0.30	−0.39	−0.22
Burnout: physical exhaustion (T2) ~~ Burnout cognitive weariness T2	0.16	0.12	0.21
Burnout: physical exhaustion (T2) ~~ Burnout emotional exhaustion T2	0.17	0.11	0.22
Burnout: physical exhaustion (T2) ~~ PWA: instructional management (T2)	−0.16	−0.21	−0.10
Burnout: physical exhaustion (T2) ~~ PWA: navigating difficult situations (T2)	−0.18	−0.23	−0.13
Burnout: physical exhaustion (T2) ~~ PWA: non-teaching responsibilities (T2)	−0.16	−0.21	−0.11
Burnout: physical exhaustion (T2) ~~ PWA teacher-staff interaction T2	−0.13	−0.18	−0.08
Burnout: physical exhaustion (T2) ~~ PWA: teaching organization (T2)	−0.17	−0.23	−0.12
Gender ~~ Age	0.13	0.04	0.22
Gender ~~ Years of practice	0.15	0.06	0.24
PWA: instructional management (T1) ~~ PWA: navigating difficult situations (T1)	0.68	0.58	0.79
PWA: instructional management (T1) ~~ PWA: non-teaching responsibilities (T1)	0.72	0.62	0.83
PWA: instructional management (T1) ~~ PWA: teacher-staff interaction (T1)	0.72	0.62	0.82
PWA: instructional management (T2) ~~ PWA: navigating difficult situations (T2)	0.49	0.43	0.57
PWA: instructional management (T2) ~~ PWA: non-teaching responsibilities (T2)	0.54	0.47	0.62
PWA: instructional management (T2) ~~ PWA teacher-staff interaction T2	0.46	0.40	0.54
PWA: navigating difficult situations (T1) ~~ PWA: non-teaching responsibilities (T1)	0.76	0.67	0.88
PWA: navigating difficult situations (T2) ~~ PWA: non-teaching responsibilities (T2)	0.52	0.45	0.59
PWA: teacher-staff interaction (T1) ~~ PWA: navigating difficult situations (T1)	0.79	0.69	0.90
PWA: teacher-staff interaction (T1) ~~ PWA: non-teaching responsibilities (T1)	0.79	0.69	0.90
PWA: teacher-staff interaction (T2) ~~ PWA: navigating difficult situations (T2)	0.45	0.39	0.53
PWA: teacher-staff interaction (T2) ~~ PWA: non-teaching responsibilities (T2)	0.56	0.49	0.64

PWA: teaching organization (T1) ~~ PWA: instructional management (T1)	0.73	0.63	0.84
PWA: teaching organization (T1) ~~ PWA: navigating difficult situations (T1)	0.65	0.55	0.75
PWA: teaching organization (T1) ~~ PWA: non-teaching responsibilities (T1)	0.63	0.53	0.73
PWA: teaching organization (T1) ~~ PWA: teacher-staff interaction (T1)	0.71	0.62	0.82
PWA: teaching organization (T2) ~~ PWA: instructional management (T2)	0.59	0.51	0.67
PWA: teaching organization (T2) ~~ PWA: navigating difficult situations (T2)	0.49	0.43	0.57
PWA: teaching organization (T2) ~~ PWA: non-teaching responsibilities (T2)	0.54	0.47	0.62
PWA: teaching organization (T2) ~~ PWA teacher-staff interaction T2	0.48	0.41	0.55
**Intercepts**			
Intercept: Age	0.00	−0.09	0.09
Intercept: Burnout: cognitive weariness (T1)	0.00	−0.09	0.09
Intercept: Burnout cognitive weariness T2	0.00	−0.06	0.06
Intercept: Burnout: emotional exhaustion (T1)	0.00	−0.09	0.09
Intercept: Burnout emotional exhaustion T2	0.00	−0.07	0.07
Intercept: Burnout: physical exhaustion (T1)	0.00	−0.09	0.09
Intercept: Burnout: physical exhaustion (T2)	0.00	−0.06	0.06
Intercept: Gender	0.00	−0.09	0.09
Intercept: PWA: instructional management (T1)	0.00	−0.09	0.09
Intercept: PWA: instructional management (T2)	0.00	−0.07	0.07
Intercept: PWA: navigating difficult situations (T1)	0.00	−0.09	0.09
Intercept: PWA: navigating difficult situations (T2)	0.00	−0.07	0.07
Intercept: PWA: non-teaching responsibilities (T1)	0.00	−0.09	0.09
Intercept: PWA: non-teaching responsibilities (T2)	0.00	−0.07	0.07
Intercept: PWA: teacher-staff interaction (T1)	0.00	−0.09	0.09
Intercept: PWA teacher-staff interaction T2	0.00	−0.07	0.07
Intercept: PWA: teaching organization (T1)	0.00	−0.09	0.09
Intercept: PWA: teaching organization (T2)	0.00	−0.07	0.07
Intercept: Years of practice	0.00	−0.09	0.09
**Variances**			
Variance: Age	1.00	0.88	1.14
Variance: Burnout: cognitive weariness (T1)	0.98	0.87	1.11
Variance: Burnout cognitive weariness T2	0.43	0.38	0.49
Variance: Burnout: emotional exhaustion (T1)	0.99	0.88	1.12
Variance: Burnout emotional exhaustion T2	0.62	0.54	0.70
Variance: Burnout: physical exhaustion (T1)	0.99	0.88	1.12
Variance: Burnout: physical exhaustion (T2)	0.49	0.43	0.56
Variance: Gender	1.01	0.89	1.15
Variance: PWA: instructional management (T1)	0.96	0.85	1.09
Variance: PWA: instructional management (T2)	0.68	0.60	0.77
Variance: PWA: navigating difficult situations (T1)	0.95	0.84	1.07
Variance: PWA: navigating difficult situations (T2)	0.60	0.52	0.68
Variance: PWA: non-teaching responsibilities (T1)	0.97	0.86	1.10
Variance: PWA: non-teaching responsibilities (T2)	0.63	0.56	0.71
Variance: PWA: teacher-staff interaction (T1)	0.94	0.83	1.06
Variance: PWA teacher-staff interaction T2	0.64	0.57	0.73
Variance: PWA: teaching organization (T1)	0.96	0.85	1.09
Variance: PWA: teaching organization (T2)	0.66	0.58	0.75
Variance: Years of practice	1.00	0.88	1.14

Supplementary Table S3. Fit indices

	T1					T2				
	**α**	**CFI**	**TLI**	**RMSEA**	**SRMR**	**α**	**CFI**	**TLI**	**RMSEA**	**SRMR**
**PWA**	0.95	0.92	0.91	0.08	0.05	0.95	0.94	0.93	0.08	0.05
teaching organization	0.91					0.93				
instructional management	0.89					0.90				
teacher-staff interaction	0.86					0.89				
navigating difficult situations	0.90					0.94				
non-teaching responsibilities	0.84					0.86				
**Burnout**	0.93	0.96	0.95	0.07	0.04	0.93	0.95	0.94	0.09	0.05
physical exhaustion	0.92					0.93				
cognitive weariness	0.92					0.93				
emotional exhaustion	0.88					0.90				

Supplementary Table S4. Imputed model results – without factors.

Effect name	M	CI_lower	CI_upper	PD
**Cross-lagged relations**				
PWA (T2)~Burnout (T1)	−0.24	−0.36	−0.13	1.00
Burnout (T2)~PWA (T1)	0.19	0.10	0.25	1.00
**Autoregressive effects**				
Burnout (T2)~Burnout (T1)	1.06	1.00	1.10	1.00
PWA (T2)~PWA (T1)	0.49	0.39	0.58	1.00
**Control effects**				
Burnout (T2)~Gender	0.03	−0.03	0.09	0.81
PWA (T2)~Gender	−0.09	−0.26	0.09	0.84
Burnout (T1)~Gender	0.15	−0.06	0.35	0.92
PWA T1~Gender	−0.02	−0.21	0.18	0.57
Burnout (T2)~Age	0.00	−0.04	0.04	0.51
PWA (T2)~Age	0.01	−0.13	0.13	0.54
Burnout (T1)~Age	−0.09	−0.24	0.06	0.89
PWA (T1)~Age	0.01	−0.13	0.16	0.57
Burnout (T2)~Years of practice	−0.04	−0.08	0.00	0.96
PWA (T2)~Years of practice	0.12	−0.01	0.25	0.96
Burnout (T1)~Years of practice	−0.02	−0.16	0.13	0.58
PWA (T1)~Years of practice	0.16	0.02	0.31	0.99
**Covariances**				
Burnout (T2)~~PWA (T2)	−0.17	−0.22	−0.11	1.00
Burnout (T1)~~PWA (T1)	−0.55	−0.63	−0.47	1.00
Gender~~Age	0.04	0.02	0.06	1.00
Gender~~Years of practice	0.04	0.01	0.06	1.00
Age~~Years of practice	0.89	0.80	0.98	1.00
Burnout (T2)~~Burnout (T2)	0.06	0.03	0.09	1.00
PWA (T2)~~PWA (T2)	0.53	0.46	0.61	1.00
Burnout (T1)~~Burnout (T1)	0.99	0.90	1.09	1.00
PWA (T1)~~PWA (T1)	0.97	0.89	1.07	1.00
Gender~~Gender	0.12	0.11	0.13	1.00
Age~~Age	1.00	0.91	1.10	1.00
Years of practice~~Years of practice	1.00	0.91	1.10	1.00

Supplementary Table S5. Imputed model results – with factors.

Effect_name	M	CI_lower	CI_upper	PD
**Cross-lagged relations**				
PWA: teaching organization (T2) ~ Burnout: physical exhaustion (T1)	−0.10	−0.21	0.01	0.96
PWA: teaching organization (T2) ~ Burnout: cognitive weariness (T1)	−0.12	−0.24	−0.01	0.98
PWA: teaching organization (T2) ~ Burnout: emotional exhaustion (T1)	−0.04	−0.14	0.04	0.83
PWA: instructional management (T2) ~ Burnout: physical exhaustion (T1)	−0.09	−0.19	0.02	0.94
PWA: instructional management (T2) ~ Burnout: cognitive weariness (T1)	−0.11	−0.21	0.01	0.96
PWA: instructional management (T2) ~ Burnout: emotional exhaustion (T1)	−0.07	−0.17	0.02	0.95
PWA: teacher-staff interaction (T2) ~ Burnout: physical exhaustion (T1)	−0.05	−0.17	0.06	0.81
PWA: teacher-staff interaction (T2) ~ Burnout: cognitive weariness (T1)	−0.06	−0.17	0.05	0.86
PWA: teacher-staff interaction (T2) ~ Burnout: emotional exhaustion (T1)	−0.13	−0.22	−0.04	1.00
PWA: navigating difficult situations (T2) ~ Burnout: physical exhaustion (T1)	−0.13	−0.24	−0.04	1.00
PWA: navigating difficult situations (T2) ~ Burnout: cognitive weariness (T1)	−0.06	−0.16	0.04	0.85
PWA: navigating difficult situations (T2) ~ Burnout: emotional exhaustion (T1)	−0.05	−0.13	0.03	0.90
PWA: non-teaching responsibilities (T2) ~ Burnout: physical exhaustion (T1)	−0.09	−0.20	0.01	0.96
PWA: non-teaching responsibilities (T2) ~ Burnout: cognitive weariness (T1)	−0.08	−0.19	0.02	0.94
PWA: non-teaching responsibilities (T2) ~ Burnout: emotional exhaustion (T1)	−0.12	−0.21	−0.04	1.00
Burnout: physical exhaustion (T2) ~ PWA: teaching organization (T1)	0.13	0.04	0.23	1.00
Burnout: physical exhaustion (T2) ~ PWA: instructional management (T1)	0.03	−0.09	0.15	0.72
Burnout: physical exhaustion (T2) ~ PWA: teacher-staff interaction (T1)	−0.01	−0.16	0.16	0.53
Burnout: physical exhaustion (T2) ~ PWA: navigating difficult situations (T1)	−0.03	−0.17	0.11	0.64
Burnout: physical exhaustion (T2) ~ PWA: non-teaching responsibilities (T1)	−0.05	−0.20	0.07	0.78
Burnout: cognitive weariness (T2) ~ PWA: teaching organization (T1)	−0.02	−0.11	0.07	0.69
Burnout: cognitive weariness (T2) ~ PWA: instructional management (T1)	0.02	−0.13	0.14	0.62
Burnout: cognitive weariness (T2) ~ PWA: teacher-staff interaction (T1)	0.05	−0.07	0.19	0.77
Burnout: cognitive weariness (T2) ~ PWA: navigating difficult situations (T1)	0.05	−0.07	0.15	0.79
Burnout: cognitive weariness (T2) ~ PWA: non-teaching responsibilities (T1)	−0.01	−0.12	0.10	0.59
Burnout: emotional exhaustion (T2) ~ PWA: teaching organization (T1)	0.05	−0.05	0.16	0.84
Burnout: emotional exhaustion (T2) ~ PWA: instructional management (T1)	−0.07	−0.22	0.09	0.80
Burnout: emotional exhaustion (T2) ~ PWA: teacher-staff interaction (T1)	−0.02	−0.17	0.13	0.60
Burnout: emotional exhaustion (T2) ~ PWA: navigating difficult situations (T1)	0.07	−0.09	0.22	0.80
Burnout: emotional exhaustion (T2) ~ PWA: non-teaching responsibilities (T1)	−0.01	−0.14	0.14	0.58
**Autoregressive effects**				
Burnout: physical exhaustion (T2) ~ Burnout: physical exhaustion (T1)	0.64	0.57	0.71	1.00
Burnout: cognitive weariness (T2) ~ Burnout: cognitive weariness (T1)	0.72	0.66	0.78	1.00
Burnout: emotional exhaustion (T2) ~ Burnout: emotional exhaustion (T1)	0.55	0.47	0.62	1.00
PWA: teaching organization (T2) ~ PWA: teaching organization (T1)	0.21	0.16	0.26	1.00
PWA: instructional management (T2) ~ PWA: instructional management (T1)	0.22	0.16	0.27	1.00
PWA: teacher-staff interaction (T2) ~ PWA: teacher-staff interaction (T1)	0.23	0.19	0.28	1.00
PWA: navigating difficult situations (T2) ~ PWA: navigating difficult situations (T1)	0.30	0.24	0.35	1.00
PWA: non-teaching responsibilities (T2) ~ PWA: non-teaching responsibilities (T1)	0.22	0.19	0.26	1.00
**Control effects**				
Burnout: physical exhaustion (T2) ~ Gender	0.17	−0.01	0.35	0.97
Burnout: cognitive weariness (T2) ~ Gender	−0.05	−0.23	0.12	0.70
Burnout: emotional exhaustion (T2) ~ Gender	0.00	−0.24	0.21	0.50
PWA: teaching organization (T2) ~ Gender	−0.10	−0.30	0.09	0.84

PWA: instructional management (T2) ~ Gender	−0.11	−0.32	0.11	0.84
PWA: teacher-staff interaction (T2) ~ Gender	−0.12	−0.34	0.10	0.85
PWA: navigating difficult situations (T2) ~ Gender	−0.11	−0.29	0.08	0.86
PWA: non-teaching responsibilities (T2) ~ Gender	−0.12	−0.31	0.09	0.87
Burnout: physical exhaustion (T1) ~ Gender	0.25	0.05	0.44	0.99
Burnout: cognitive weariness (T1) ~ Gender	0.20	0.01	0.40	0.98
Burnout: emotional exhaustion (T1) ~ Gender	−0.17	−0.36	0.03	0.95
PWA: teaching organization (T1) ~ Gender	0.16	−0.03	0.35	0.95
PWA: instructional management (T1) ~ Gender	0.05	−0.14	0.24	0.70
PWA: teacher-staff interaction (T1) ~ Gender	−0.06	−0.25	0.13	0.75
PWA: navigating difficult situations (T1) ~ Gender	0.02	−0.18	0.21	0.56
PWA: non-teaching responsibilities (T1) ~ Gender	−0.09	−0.28	0.10	0.83
Burnout: physical exhaustion (T2) ~ Age	−0.08	−0.20	0.04	0.91
Burnout: cognitive weariness (T2) ~ Age	0.03	−0.09	0.16	0.71
Burnout: emotional exhaustion (T2) ~ Age	0.02	−0.12	0.15	0.59
PWA: teaching organization (T2) ~ Age	0.00	−0.15	0.16	0.53
PWA: instructional management (T2) ~ Age	0.00	−0.16	0.14	0.51
PWA: teacher-staff interaction (T2) ~ Age	0.03	−0.12	0.17	0.66
PWA: navigating difficult situations (T2) ~ Age	−0.01	−0.16	0.14	0.51
PWA: non-teaching responsibilities (T2) ~ Age	0.02	−0.13	0.16	0.61
Burnout: physical exhaustion (T1) ~ Age	−0.14	−0.28	0.01	0.97
Burnout: cognitive weariness (T1) ~ Age	−0.10	−0.24	0.05	0.91
Burnout: emotional exhaustion (T1) ~ Age	−0.03	−0.18	0.12	0.66
PWA: teaching organization (T1) ~ Age	0.06	−0.08	0.20	0.79
PWA: instructional management (T1) ~ Age	−0.03	−0.17	0.11	0.65
PWA: teacher-staff interaction (T1) ~ Age	−0.01	−0.15	0.13	0.55
PWA: navigating difficult situations (T1) ~ Age	0.02	−0.12	0.16	0.60
PWA: non-teaching responsibilities (T1) ~ Age	0.01	−0.14	0.15	0.54
Burnout: physical exhaustion (T2) ~ Years_of_practice	−0.01	−0.12	0.11	0.54
Burnout: cognitive weariness (T2) ~ Years_of_practice	−0.07	−0.19	0.04	0.89
Burnout: emotional exhaustion (T2) ~ Years_of_practice	−0.07	−0.20	0.07	0.83
PWA: teaching organization (T2) ~ Years_of_practice	0.16	0.01	0.32	0.98
PWA: instructional management (T2) ~ Years_of_practice	0.16	0.02	0.30	0.99
PWA: teacher-staff interaction (T2) ~ Years_of_practice	0.17	0.03	0.33	0.99
PWA: navigating difficult situations (T2) ~ Years_of_practice	0.13	−0.01	0.28	0.97
PWA: non-teaching responsibilities (T2) ~ Years_of_practice	0.18	0.04	0.33	0.99
Burnout: physical exhaustion (T1) ~ Years_of_practice	0.07	−0.07	0.22	0.84
Burnout: cognitive weariness (T1) ~ Years_of_practice	0.01	−0.14	0.15	0.53
Burnout: emotional exhaustion (T1) ~ Years_of_practice	−0.02	−0.17	0.12	0.62
PWA: teaching organization (T1) ~ Years_of_practice	0.09	−0.05	0.24	0.90
PWA: instructional management (T1) ~ Years_of_practice	0.18	0.04	0.32	0.99
PWA: teacher-staff interaction (T1) ~ Years_of_practice	0.19	0.05	0.33	1.00
PWA: navigating difficult situations (T1) ~ Years_of_practice	0.15	0.00	0.29	0.98
PWA: non-teaching responsibilities (T1) ~ Years_of_practice	0.09	−0.06	0.23	0.88
**Covariances**				
Gender ~~ Age	0.16	0.11	0.22	1.00
Gender ~~ Gender	0.18	0.11	0.23	1.00
Gender ~~ Years_of_practice	−0.20	−0.27	−0.12	1.00

PWA: instructional management (T1) ~ Age	−0.18	−0.24	−0.11	1.00
PWA: instructional management (T1) ~ Gender	−0.15	−0.22	−0.09	1.00
PWA: instructional management (T1) ~ Years_of_practice	−0.20	−0.26	−0.14	1.00
PWA: instructional management (T1) ~~ PWA: instructional management (T1)	−0.18	−0.25	−0.12	1.00
PWA: instructional management (T1) ~~ PWA: navigating difficult situations (T1)	0.24	0.18	0.31	1.00
PWA: instructional management (T1) ~~ PWA: non-teaching responsibilities (T1)	−0.15	−0.21	−0.09	1.00
PWA: instructional management (T1) ~~ PWA: teacher-staff interaction (T1)	−0.14	−0.20	−0.08	1.00
PWA: instructional management (T2) ~ Age	−0.13	−0.19	−0.07	1.00
PWA: instructional management (T2) ~ Gender	−0.14	−0.20	−0.08	1.00
PWA: instructional management (T2) ~ PWA: instructional management (T1)	−0.14	−0.20	−0.08	1.00
PWA: instructional management (T2) ~ Years_of_practice	−0.19	−0.26	−0.11	1.00
PWA: instructional management (T2) ~~ PWA: instructional management (T2)	−0.17	−0.25	−0.08	1.00
PWA: instructional management (T2) ~~ PWA: navigating difficult situations (T2)	−0.17	−0.25	−0.10	1.00
PWA: instructional management (T2) ~~ PWA: non-teaching responsibilities (T2)	−0.15	−0.23	−0.08	1.00
PWA: instructional management (T2) ~~ PWA teacher-staff interaction T2	−0.17	−0.24	−0.09	1.00
PWA: navigating difficult situations (T1) ~ Age	0.62	0.55	0.71	1.00
PWA: navigating difficult situations (T1) ~ Gender	0.52	0.45	0.59	1.00
PWA: navigating difficult situations (T1) ~ Years_of_practice	0.54	0.47	0.62	1.00
PWA: navigating difficult situations (T1) ~~ PWA: navigating difficult situations (T1)	0.59	0.52	0.66	1.00
PWA: navigating difficult situations (T1) ~~ PWA: non-teaching responsibilities (T1)	0.50	0.43	0.58	1.00
PWA: navigating difficult situations (T2) ~ Age	0.53	0.46	0.61	1.00
PWA: navigating difficult situations (T2) ~ Gender	0.58	0.51	0.66	1.00
PWA: navigating difficult situations (T2) ~ PWA: navigating difficult situations (T1)	0.49	0.42	0.56	1.00
PWA: navigating difficult situations (T2) ~ Years_of_practice	0.59	0.52	0.67	1.00
PWA: navigating difficult situations (T2) ~~ PWA: navigating difficult situations (T2)	0.55	0.48	0.62	1.00
PWA: navigating difficult situations (T2) ~~ PWA: non-teaching responsibilities (T2)	0.66	0.59	0.75	1.00
PWA: non-teaching responsibilities (T1) ~ Age	0.56	0.48	0.64	1.00
PWA: non-teaching responsibilities (T1) ~ Gender	−0.32	−0.39	−0.25	1.00
PWA: non-teaching responsibilities (T1) ~ Years_of_practice	−0.29	−0.35	−0.22	1.00
PWA: non-teaching responsibilities (T1) ~~ PWA: non-teaching responsibilities (T1)	−0.36	−0.43	−0.29	1.00
PWA: non-teaching responsibilities (T2) ~ Age	−0.39	−0.46	−0.32	1.00
PWA: non-teaching responsibilities (T2) ~ Gender	−0.35	−0.42	−0.28	1.00
PWA: non-teaching responsibilities (T2) ~ PWA: non-teaching responsibilities (T1)	0.60	0.52	0.68	1.00
PWA: non-teaching responsibilities (T2) ~ Years_of_practice	−0.35	−0.42	−0.28	1.00
PWA: non-teaching responsibilities (T2) ~~ PWA: non-teaching responsibilities (T2)	−0.35	−0.42	−0.29	1.00
PWA: teacher-staff interaction (T1) ~ Age	−0.37	−0.44	−0.30	1.00
PWA: teacher-staff interaction (T1) ~ Gender	−0.37	−0.44	−0.30	1.00
PWA: teacher-staff interaction (T1) ~ Years_of_practice	−0.36	−0.43	−0.29	1.00
PWA: teacher-staff interaction (T1) ~~ PWA: navigating difficult situations (T1)	−0.33	−0.40	−0.26	1.00
PWA: teacher-staff interaction (T1) ~~ PWA: non-teaching responsibilities (T1)	−0.30	−0.37	−0.23	1.00
PWA: teacher-staff interaction (T1) ~~ PWA: teacher-staff interaction (T1)	−0.34	−0.41	−0.28	1.00
PWA: teacher-staff interaction (T2) ~ Age	−0.34	−0.41	−0.28	1.00
PWA: teacher-staff interaction (T2) ~ Gender	−0.33	−0.40	−0.27	1.00
PWA: teacher-staff interaction (T2) ~ PWA: teacher-staff interaction (T1)	0.72	0.65	0.81	1.00
PWA: teacher-staff interaction (T2) ~ Years_of_practice	0.70	0.63	0.79	1.00
PWA: teacher-staff interaction (T2) ~~ PWA: navigating difficult situations (T2)	0.64	0.57	0.72	1.00
PWA: teacher-staff interaction (T2) ~~ PWA: non-teaching responsibilities (T2)	0.64	0.56	0.72	1.00
PWA: teacher-staff interaction (T2) ~~ PWA teacher-staff interaction T2	0.72	0.65	0.81	1.00

PWA: teaching organization (T1) ~ Age	0.68	0.60	0.76	1.00
PWA: teaching organization (T1) ~ Gender	0.74	0.67	0.83	1.00
PWA: teaching organization (T1) ~ Years_of_practice	0.80	0.72	0.89	1.00
PWA: teaching organization (T1) ~~ PWA: instructional management (T1)	0.79	0.71	0.88	1.00
PWA: teaching organization (T1) ~~ PWA: navigating difficult situations (T1)	0.77	0.69	0.85	1.00
PWA: teaching organization (T1) ~~ PWA: non-teaching responsibilities (T1)	0.04	0.02	0.07	1.00
PWA: teaching organization (T1) ~~ PWA: teacher-staff interaction (T1)	0.04	0.01	0.06	1.00
PWA: teaching organization (T1) ~~ PWA: teaching organization (T1)	0.89	0.80	0.98	1.00
PWA: teaching organization (T2) ~ Age	0.49	0.43	0.57	1.00
PWA: teaching organization (T2) ~ Gender	0.43	0.37	0.50	1.00
PWA: teaching organization (T2) ~ PWA: teaching organization (T1)	0.62	0.55	0.71	1.00
PWA: teaching organization (T2) ~ Years_of_practice	0.69	0.62	0.77	1.00
PWA: teaching organization (T2) ~~ PWA: instructional management (T2)	0.70	0.62	0.78	1.00
PWA: teaching organization (T2) ~~ PWA: navigating difficult situations (T2)	0.68	0.60	0.75	1.00
PWA: teaching organization (T2) ~~ PWA: non-teaching responsibilities (T2)	0.62	0.55	0.69	1.00
PWA: teaching organization (T2) ~~ PWA teacher-staff interaction T2	0.66	0.59	0.74	1.00
PWA: teaching organization (T2) ~~ PWA: teaching organization (T2)	0.99	0.90	1.09	1.00
Years_of_practice ~~ Years_of_practice	0.99	0.90	1.08	1.00
Gender ~~ Age	0.99	0.90	1.09	1.00
Gender ~~ Gender	0.96	0.88	1.06	1.00
Gender ~~ Years_of_practice	0.96	0.88	1.06	1.00
PWA: instructional management (T1) ~ Age	0.95	0.87	1.05	1.00
PWA: instructional management (T1) ~ Gender	0.96	0.87	1.06	1.00
PWA: instructional management (T1) ~ Years_of_practice	0.98	0.89	1.07	1.00
PWA: instructional management (T1) ~~ PWA: instructional management (T1)	0.12	0.11	0.13	1.00
PWA: instructional management (T1) ~~ PWA: navigating difficult situations (T1)	1.00	0.91	1.10	1.00
PWA: instructional management (T1) ~~ PWA: non-teaching responsibilities (T1)	1.00	0.91	1.10	1.00
